# Taxation of the fat content of foods for reducing their consumption and preventing obesity or other adverse health outcomes

**DOI:** 10.1002/14651858.CD012415.pub2

**Published:** 2020-09-11

**Authors:** Stefan K Lhachimi, Frank Pega, Thomas L Heise, Candida Fenton, Gerald Gartlehner, Ursula Griebler, Isolde Sommer, Manuela Bombana, Srinivasa Vittal Katikireddi

**Affiliations:** Research Group for Evidence-Based Public HealthLeibniz Institute for Prevention Research and EpidemiologyBremenGermany; Public HealthUniversity of OtagoWellingtonNew Zealand; Institute for Public Health and Nursing Research, Health Sciences BremenUniversity of BremenBremenGermany; Usher Institute of Population Health Sciences and InformaticsUniversity of EdinburghEdinburghUK; Cochrane Austria, Department for Evidence-based Medicine and EvaluationDanube University KremsKremsAustria; Department of Health PromotionAOK Baden-WürttembergStuttgartGermany; MRC/CSO Social and Public Health Sciences UnitUniversity of GlasgowGlasgowUK; Department for Health Services Research, Institute for Public Health and Nursing Research, Health Sciences BremenUniversity of BremenBremenGermany; RTI InternationalResearch Triangle ParkNorth CarolinaUSA; Department of General Practice and Health Services ResearchUniversity Hospital, University of HeidelbergHeidelbergGermany

## Abstract

**Background:**

Overweight and obesity are increasing worldwide and are considered to be a major public health issue of the 21st century. Introducing taxation of the fat content in foods is considered a potentially powerful policy tool to reduce consumption of foods high in fat or saturated fat, or both.

**Objectives:**

To assess the effects of taxation of the fat content in food on consumption of total fat and saturated fat, energy intake, overweight, obesity, and other adverse health outcomes in the general population.

**Search methods:**

We searched CENTRAL, Cochrane Database of Systematic Reviews, MEDLINE, Embase, and 15 other databases and trial registers on 12 September 2019. We handsearched the reference lists of all records of included studies, searched websites of international organizations and institutions (14 October 2019), and contacted review advisory group members to identify planned, ongoing, or unpublished studies (26 February 2020).

**Selection criteria:**

In line with Cochrane Effective Practice and Organisation of Care Group (EPOC) criteria, we included the following study types: randomized controlled trials (RCTs), cluster‐randomized controlled trials (cRCTs), non‐randomized controlled trials (nRCTs), controlled before‐after (CBA) studies, and interrupted time series studies. We included studies that evaluated the effects of taxes on the fat content in foods. Such a tax could be expressed as sales, excise, or special value added tax (VAT) on the final product or an intermediary product. Eligible interventions were taxation at any level, with no restriction on the duration or the implementation level (i.e. local, regional, national, or multinational). Eligible study populations were children (zero to 17 years) and adults (18 years or older) from any country and setting. We excluded studies that focused on specific subgroups only (e.g. people receiving pharmaceutical intervention; people undergoing a surgical intervention; ill people who are overweight or obese as a side effect, such as those with thyroiditis and depression; and people with chronic illness). Primary outcomes were total fat consumption, consumption of saturated fat, energy intake through fat, energy intake through saturated fat, total energy intake, and incidence/prevalence of overweight or obesity. We did not exclude studies based on country, setting, comparison, or population.

**Data collection and analysis:**

We used standard Cochrane methods for all phases of the review. Risk of bias of the included studies was assessed using the criteria of Cochrane’s ‘Risk of bias’ tool and the EPOC Group’s guidance. Results of the review are summarized narratively and the certainty of the evidence was assessed using the GRADE approach. These steps were done by two review authors, independently.

**Main results:**

We identified 23,281 records from searching electronic databases and 1173 records from other sources, leading to a total of 24,454 records. Two studies met the criteria for inclusion in the review. Both included studies investigated the effect the Danish tax on saturated fat contained in selected food items between 2011 and 2012. Both studies used an interrupted time series design. Neither included study had a parallel control group from another geographic area. The included studies investigated an unbalanced panel of approximately 2000 households in Denmark and the sales data from a specific Danish supermarket chain (1293 stores). Therefore, the included studies did not address individual participants, and no restriction regarding age, sex, and socioeconomic characteristics were defined. We judged the overall risk of bias of the two included studies as unclear.

For the outcome total consumption of fat, a reduction of 41.8 grams per week per person in a household (P < 0.001) was estimated. For the consumption of saturated fat, one study reported a reduction of 4.2% from minced beef sales, a reduction of 5.8% from cream sales, and an increase of 0.5% to sour cream sales (no measures of statistical precision were reported for these estimates). These estimates are based on a restricted number of food types and derived from sales data; they do not measure individual intake. Moreover, these estimates do not account for other relevant sources of fat intake (e.g. packaged or processed food) or other food outlets (e.g. restaurants or cafeterias); hence, we judged the evidence on the effect of taxation on total fat consumption or saturated fat consumption to be very uncertain. We did not identify evidence on the effect of the intervention on energy intake or the incidence or prevalence of overweight or obesity.

**Authors' conclusions:**

Given the very low quality of the evidence currently available, we are unable to reliably establish whether a tax on total fat or saturated fat is effective or ineffective in reducing consumption of total fat or saturated fat. There is currently no evidence on the effect of a tax on total fat or saturated fat on total energy intake or energy intake through saturated fat or total fat, or preventing the incidence or reducing the prevalence of overweight or obesity.

## Plain language summary

**Does a tax on the fat content of foods reduce obesity and help people to eat less fat and less saturated fat?**

**What is obesity?**

Body mass index (BMI) is usually a useful measure to show whether people are a healthy weight for their height. A BMI of 18 to 25 shows a healthy weight, a BMI over 25 is considered overweight, and a BMI over 30 shows obesity.

Obesity is generally caused by consuming more energy (calories) than you burn off through physical activity or exercise. The body stores the excess energy as fat.

Obesity can lead to serious and life‐threatening conditions, including type 2 diabetes, coronary heart disease, some types of cancer, and stroke. Obesity can also affect people's quality of life (well‐being).

**What are fats?**

Fat is essential to a healthy, balanced diet. Fats help the body to absorb some vitamins, and are a source of some fatty acids that the body cannot make. The main types of fat in foods are:

‐ saturated (mostly from animal products, such as meat, cow's milk, cheese, cream, and butter); and

‐ unsaturated (mostly found in oils from plants and fish).

Health guidelines usually recommend that people should:

‐ reduce the overall amount of fat they eat;

‐ limit the amount of saturated fats they eat; and

‐ eat unsaturated fats instead of saturated fats.

**Why we did this Cochrane Review**

Rates of overweight and obesity are increasing around the world and are a major public health issue. We wanted to know whether increasing the prices of foods that have a lot of fats or saturated fats could put people off eating them, and reduce obesity.

**What did we do?**

We searched for studies that looked at the effects of a tax on the fat content of foods. We were interested in how a tax affected the:

‐ total amount of fat, and saturated fats eaten;

‐ total calories eaten;

‐ calories eaten from all fats and saturated fats; and

‐ rates of overweight and obesity.

**Search date:** we included evidence published up to September 2019.

**What we found**

We found two studies from Denmark, conducted during 2011 to 2012. One looked at how a tax on some high‐fat foods affected household demand for them; the other looked at information on supermarket sales for certain high‐fat foods (minced beef, cream, and sour cream). They compared their results with data from before the tax started.

Both studies looked at a small number of foods that people bought, but not what foods people ate. They didn't measure how much total fat or saturated fat were eaten.

**What are the results of our review?**

If the amount of foods bought reflected the amount of foods eaten, then taxing the fat content of certain foods:

‐ might reduce the total amount of fats eaten by 41.8 grams a week for each person in a household, in one study of 2000 households; and

‐ might reduce the amount of saturated fats eaten (in minced beef and cream), in one study of 1293 supermarkets.

No studies measured the effect of taxing the fat content of foods on calories eaten, on obesity or overweight, or on total food sales.

**How reliable are these results?**

We are not confident in the results because the evidence is only from two studies; and these studies only measured a small number of foods bought, and did not measure foods eaten. One study did not report statistics about the accuracy of its results.

The results were from observational studies, in which researchers observe the effect of a factor (such as taxation) without trying to change who does, or does not, experience it. Observational studies do not give as reliable evidence as randomized controlled studies, in which the treatments people receive are decided at random.

**Conclusions**

We did not find enough reliable evidence to find out whether a tax on the fat content of foods resulted in people eating less fat, or less saturated fat.

We did not find any evidence about how a tax on the fat content of foods affected obesity or overweight.

The results of our review will change when further evidence becomes available.

## Summary of findings

**Summary of findings 1:** Taxation of the fat content of foods compared to no taxation for reducing their consumption and preventing obesity or other adverse health outcomes

**Taxation of the fat content of foods compared to no taxation for reducing their consumption and preventing obesity or other adverse health outcomes**			
**Patient or population:** general population of Denmark **Setting:** Denmark **Intervention:** taxation of the fat content of foods **Comparison:** no taxation			
**Outcomes**	**№ of participants (studies) Follow up**	**Certainty of the evidence (GRADE)**	**Impact**
Total fat consumption	2000 households (ITS design) (1 observational study)	⊕⊝⊝⊝ VERY LOW ^1 2^	There is very uncertain evidence that taxing the fat content of foods reduces estimated total fat consumption by 41.8 grams per week, per person in a household (P < 0.001).
Total saturated fat consumption	1293 supermarkets (ITS design) (1 observational study)	⊕⊝⊝⊝ VERY LOW ^1 2 3^	There is very uncertain evidence that taxing the fat content of foods reduces the estimated saturated fat content of sales by 4.2% for minced beef and by 5.8% for cream, and increases the estimated saturated fat content of sales by 0.5% for sour cream. (No measure of statistical precision was reported for any of these results.)
Energy intake	See comment	0 (0)	Not reported^4^
Overweight	See comment	0 (0)	Not reported^4^
Obesity	See comment	0 (0)	Not reported^4^
Total sales	See comment	0 (0)	Not reported^4^
ITS: interrupted time series			
**GRADE Working Group grades of evidence** **High certainty:** we are very confident that the true effect lies close to that of the estimate of the effect **Moderate certainty:** we are moderately confident in the effect estimate: The true effect is likely to be close to the estimate of the effect, but there is a possibility that it is substantially different **Low certainty:** our confidence in the effect estimate is limited: The true effect may be substantially different from the estimate of the effect **Very low certainty:** we have very little confidence in the effect estimate: The true effect is likely to be substantially different from the estimate of effect			

^1^ Observational studies with an ITS design start out at the level 'low‐certainty'. ^2^ Downgraded 2 levels because of indirectness (calculation of effect estimates were based on a restricted number of food items and sales/purchases of food items was used as a proxy to measure consumption). ^3^ Downgraded 2 levels because of imprecision (study did not report confidence intervals, P values or any other measure about the statistical precision of the effect estimates). ^4^ No study measured the effects of taxing fat content of food on energy intake, overweight, obesity, or total sales.

## Background

### Description of the condition

Overweight and obesity (a body mass index (BMI) of 25 or more and 30 or more, respectively) are increasing worldwide and present a major public health issue of the 21st century (NCD‐RisC 2016; WHO 2014; Swinburn 2019). The Global Burden of Disease Study estimated that the prevalence of obesity more than doubled between 1980 and 2017 (GBD 2017). In 2016, approximately 2 billion adults were overweight, and of these over 650 million were obese; that is, 39% of adults aged 18 years and over were overweight in 2016, and 13% were obese. Similarly, approximately 340 million children and adolescents aged five to 19 were overweight or obese in 2016, and 40 million children under the age of five were overweight or obese in 2018 (WHO 2020). Although the increase of adult obesity has stabilized (albeit at very high levels) in some high‐income countries (HICs), the prevalence of obesity in low‐ and middle‐income countries (LMICs) and several HICs is continuing to rise (Ng 2014; Wells 2020). The reasons for these trends are complex and influenced by a broad variety of social determinants of health, such as urbanization, changes in types of employment, and alterations to the food supply (Lang 2009). In LMICs the rise has been partly attributed to economic modernization and lifestyle changes, i.e. a transition to a 'Western diet' that is broadly defined by a high intake of refined carbohydrates, added sugars, fats, and animal‐source foods (Goryakin 2015; Popkin 2012).

Obesity is a major risk factor for mortality and morbidity (Lhachimi 2013). In 2015, excessive body weight was estimated to cause 4 million deaths and accounted for a loss of 120 million disability‐adjusted life years (Swinburn 2019). In particular, non‐communicable diseases, such as type 2 diabetes, cardiovascular diseases, certain cancers, and musculoskeletal disorders, are potential health consequences of a raised BMI (Barber 2018; Guh 2009). This also makes obesity a significant factor for disability (Lhachimi 2016). Non‐communicable diseases are already the leading cause of death in HICs, and are increasing in LMICs (WHO 2014). Moreover, the increased prevalence of chronic diseases in regions where individuals have insufficient access to appropriate health care may exacerbate the harmful consequences of obesity on morbidity and mortality for those populations. For example, if an obese person with type 2 diabetes does not have regular access to insulin, this may result in particularly premature death, disability, or morbidity (Seidell 2015; Wells 2020).

Overweight and obesity are often defined as the "abnormal or excessive body fat accumulation in adipose tissue" (WHO 2000; WHO 2011). At the individual level, overweight and obesity are mainly caused by an imbalance in energy intake and energy expenditure. In the 2014 *Declaration of Rome on Nutrition,* the member states of the Food and Agriculture Organization (FAO) and the World Health Organization (WHO) noted certain aspects of a diet that increase the susceptibility to both overweight and obesity, as well as comorbid non‐communicable diseases; chief among these was consumption of food that is high in fat (FAO/WHO 2015). Fats are energy dense (i.e. 37 kilojoules (kJ) or 9 kilocalories **(**kcal) per gram), which contributes to the palatability of food and enables absorption of fat‐soluble vitamins. Moreover, fats are crucial for development and survival during the early stages of life, i.e. embryonic development, early growth after birth, and childhood (Burlingame 2009). Excess fat intake, however, is associated with the rise in obesity. The consumption of particular types of fat has been linked to a range of diseases and adverse health outcomes, such as type 2 diabetes, coronary heart disease, stroke, and certain types of cancer (FAO 2010; Huang 2019; Wells 2020).

Dietary fats are conventionally grouped into three broad categories based on the number of double bonds the molecules exhibit, i.e. (1) saturated fatty acids, (2) monounsaturated fatty acids, and (3) polyunsaturated fatty acids. Saturated fats are acids with only single bonds between adjacent carbon atoms, i.e. every carbon atom carries its full quota of hydrogen atoms (Bender 2014). The most notable dietary sources of saturated fats are animal products such as meat, cow's milk, eggs, butter, and salmon. Plant products, such as palm oil, coconut, and chocolate/cocoa butter, are also substantial sources of dietary saturated fat intake (Souza 2015). Unsaturated fatty acids have one or more double bonds between carbon atoms: monounsaturated fatty acids have only one of those double bonds whereas polyunsaturated fatty acids have two or more. Monounsaturated fatty acids can be found in animal and vegetable products such as red meat, dairy products, and high‐fat fruits. Many polyunsaturated fatty acids can be found in most fats, whereas certain nutritionally‐important subtypes are mostly found in oily fish such as salmon or herring (FAO 2010).

Several authoritative dietary guidelines recommend that total fat intake should contribute less than 30% of daily energy intake in adults, and that saturated fats should be limited to less than 10% of total energy intake (Eckel 2014; FAO 2010; FAO/WHO 2015; Lichtenstein 2006; NDA 2010; US Department of Agriculture 2010; WHO 2018). Hence, when reducing the total fat intake, the share of saturated fat might be lowered respectively. A systematic review (Harika 2013), however, reported that in the majority of the countries for which data were available (28 out of 45 countries), average total fat intake was above the recommended 30% energy threshold. The average proportion of energy contributed by total fats ranged from 11.1% (in Bangladesh) to 46.2% (in Greece). Moreover, for 29 countries the average saturated fat intake was larger than the recommended 10% of total energy intake, ranging from 2.9% (in Bangladesh) to 20.9% (in Indonesia). Only a few of the included studies reported data on the distribution of fat intake within a population. Notably, the share of the population with an intake above the recommended threshold varied widely between countries (e.g. approximately 95% of the Danish population has a saturated fat intake of more than 10% energy, versus only 17% of the Indian population). In particular, for LMICs the share of total fat and saturated fat intake is predicted to increase as countries develop economically and socially and, therefore, an increased intake will become a component of diets across the globe (Popkin 2020; Popkin 2012; Wolmarans 2009).

### Fat consumption and preventing obesity or other adverse health outcomes

The role of dietary fat intake in the worldwide rise in obesity is heavily debated. In particular, two major issues emerge (Bray 1998): (1) whether a decrease in overall fat intake can lead to a decrease of overweight and obesity, and (2) whether the increase of overweight and obesity in LMICs can be halted or slowed by preventing the progression towards a higher‐fat diet. A Cochrane Review (commissioned by the WHO Nutrition Guidance Expert Advisory Group (NUGAG) as part of the process of updating the guidelines on fat intake) investigated the relationship between total fat intake and obesity (Hooper 2015b). This review excluded studies that recruited populations specifically for weight loss and interventions intended to result in weight loss. Such studies are likely to be confounded by the implicit aim of reducing calorie intake, and therefore may over‐represent studies with obese populations from Western countries. This would limit the transferability to non‐obese populations or countries. Based on a meta‐analysis of the included RCTs, the review authors concluded that consuming a lower proportion of total energy from fat results in small reductions in body weight and BMI among adults. Moreover, there was no suggestion of harms that might mitigate any benefits of weight loss. These findings were confirmed in a recent update of the review (Hooper 2020).

The authors recommend that for populations where the mean total fat intake is below 30% of energy consumed, such as in many LMICs, staying below this threshold may help to avoid obesity. For populations where mean total fat intake is above the 30% energy threshold, a reduction in intake below this threshold may support the maintenance of healthy weight (Hooper 2015b). The consumption of saturated fat has long been suspected to increase the risk and incidence of coronary heart disease (Keys 1950). However, the precise relationship is still being debated. A related Cochrane Review investigated the relationship between saturated fat intake and cardiovascular disease (Hooper 2015a), and identified a robust effect on reducing combined cardiovascular events but not a general effect on all‐cause mortality or cardiovascular mortality. Regarding the association between the intake of saturated fat and type 2 diabetes, a FAO expert group from their review of the literature concluded that there is a possible positive relationship (FAO 2010), however a review solely based on observational studies did not identify such an association (Souza 2015).

One recommended alternative to reducing the total fat content of foods by lowering the total amount of saturated fat in them, is replacing saturated fat with polyunsaturated fat, as some of the latter fats may have a beneficial health effect. Saturated fats are most commonly found in processed or energy‐dense, nutrient‐poor food. The Cochrane Review suggests that replacing saturated fat with polyunsaturated fat leads to a reduction in cardiovascular events (27% less), but this is not the case for other types of replacement (e.g. with carbohydrates, protein, or monounsaturated fats) (Hooper 2015a). Similarly, a Cochrane Review investigating the effect of increasing or decreasing amounts of a certain type of polyunsaturated fat (Omega 6) did not find evidence of any beneficial or harmful effects (Al‐Khudairy 2015). Therefore, reducing the share of total energy coming from fat will have beneficial effects, while current evidence suggests that this should be predominantly achieved through a reduction in the content of saturated fat.

### Description of the intervention

Taxation as a fiscal measure is usually designed to raise revenue for government expenditure. Taxation on commodities, however, has also been used to influence consumer behaviour, e.g. taxation of foreign goods to discourage imports by making them more expensive and, hence, protecting domestic producers. Similarly, taxation has been used to generally disincentivize consumption (and production). For example, many countries are considering or already have introduced 'sin taxes' on alcohol and tobacco to prevent alcohol and tobacco use, often with the primary aim of preventing or reducing resultant public health harms (Blecher 2015). The WHO Commission on Social Determinants of Health has recommended taxation as a policy tool for addressing the social determinants of health to improve health equity (CSHD 2008). Fat taxes can be classified as an intersectoral socioeconomic intervention on the social determinants of health to improve health equity (Pega 2017).

Current evidence on the health effects of the different types of dietary fats ‐ as outlined above, and reflected in several dietary guidelines (Eckel 2014; FAO 2010; FAO/WHO 2015; Lichtenstein 2006; NDA 2010; US Department of Agriculture 2010) ‐ suggests that a tax on fat content should be designed in such a way that it may reduce the overall fat content by replacing unhealthy fats, e.g. saturated fat (WHO 2018; Schonbach 2019). In this Cochrane review, we included all types of taxation targeting fat contents in general but we paid special attention to if, and how, less desirable dietary fats (in particular saturated fats) were being affected by the intervention.

Taxation to curb the content of fat in food is usually achieved through indirect taxes, implemented either as a sales or an excise tax (Sassi 2010). While producers or sellers pay the tax to the government, they are usually expected to shift the tax burden to the consumer by raising the price of the item in question. A sales tax is usually added to the price of a product at the point of sale. Value added tax (VAT; a special form of sales tax that is very common in many European countries) avoids a taxation cascade when a product has to go through a number of intermediaries by only taxing the value added by a producer/reseller, i.e. value added equals sales price minus prices for input. The level of a sales tax can differ by type of commodity. For example, the UK has three different rates of VAT: standard (20%), reduced (5%), zero (no tax). Introducing a (higher) tax on a targeted product, e.g. foods high in saturated fat, may only require reassigning the product to a different category (Mytton 2007). A disadvantage of sales taxes/VAT, however, is that the tax is on the price and not on the volume of the product (Bonnet 2013). As larger volumes of a product are usually cheaper in relative terms than smaller volumes, the impact of a sales tax could be reduced by increasing package size. Excise taxes, on the other hand, are usually levied as a fixed rate per unit‐volume of content, independent of price or value. Hence, an excise tax may have more potential to reduce the incentives for consumers to buy larger volumes of the taxed product, or switch to cheaper brands with virtually identical fat content.

### How the intervention might work

Standard economic theory predicts that a price increase leads to a reduction in consumption. This finding, measured through elasticities, has been well established, not least for health‐relevant commodities such as tobacco and alcohol (Lhachimi 2012; Schonbach 2019a). However, it is not always clear to what extent a tax will eventually increase retail prices. Although indirect taxes are assumed to be shifted to the consumer, examples exist where producers and retailers avoided doing this fully, illustrated by calls for minimum unit pricing of alcohol as a complement to taxation (Katikireddi 2014). In addition to increasing prices paid by the consumer as a consequence of the tax, producers may broadly respond in two ways. First, taxing (excessive saturated) fat content may lead to altered production processes, resulting in lower saturated fat content in absolute terms, and thereby also reducing total fat content of a food product and the overall calorie content of a product. Second, producers may replace the share of saturated fat with other fats or nutrients, or both. Hence, the new calorie content may now be higher, lower, or unchanged. Moreover, these new ingredients may or may not have further health implications of their own. The first case is in line with the intention of such a tax and is expected to have overall beneficial health outcomes. In the second case, however, the effects of the changed food item on obesity and overall health are unclear. Similarly, the consumer may respond to tax‐induced price increases with substitution, i.e. consuming a different product. Again, the effect of this substitution on energy intake and health outcomes is uncertain (Miao 2013) and the precise nature of the substitution may strongly depend on cultural, geographical, and social factors. Price is only one determinant among other environmental, social, and cultural factors that influence consumption behaviour and individual diet (Dixon 2013). Lastly, the manner by which the intervention is introduced and implemented may impact its effectiveness. For example, taxation introduced primarily for revenue‐raising purposes may not be set at a high enough level to influence behaviour, or may not have an impact on awareness of the adverse health consequences of the product.

In Figure 1, we present a logic model showing the hypothesized causal pathways between taxation of total fat/saturated fat and obesity/other health outcomes. We anticipate that the introduction of a tax on saturated fat/total fat may influence prices or composition of food items, or both. The change in prices or composition (or both) of food items may affect buying behaviour and, in turn, food consumption. Through a change in composition or substitution (or both), the new diet may result in lower, higher, or unaltered energy intake. Similarly, the intake of total fat, saturated fat, and other nutrients will be influenced. These expected changes may have beneficial effects on obesity and other health outcomes.

**1:**
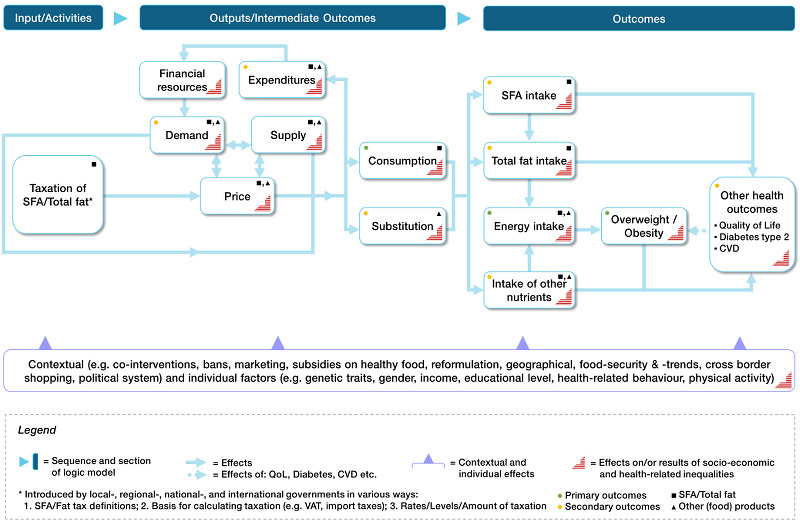
Logical model for taxation of saturated fat

Moreover, taxing a good depending on nutritional content sends a strong signal from the government to consumers and producers alike: the government is seriously concerned and is taking tangible measures to curb consumption (Sassi 2016). For example, even if the current level of taxation is low, once legislation for a tax is in place it becomes much easier to increase the tax level in the future, and the process of introducing a tax may raise awareness of the adverse health effects and facilitate behavioural change.

### Why it is important to do this review

In their global strategy on diet, physical activity and health, the World Health Assembly and the WHO stated that prices influence consumption choices and that public policies can influence prices through taxation, in ways that encourage healthy eating (Waxman 2004; WHO 2014). Moreover, taxes are considered highly cost‐effective public health actions as they may raise revenue that outstrips implementation cost (Sassi 2014). This clearly demonstrates the importance of tax interventions for public health.

The expected health effect of a tax on fat has been repeatedly suggested and analyzed in simulation studies for several countries (Jørgensen 2013; Nnoaham 2009; Thiele 2010; Tiffin 2011). Previous systematic reviews have investigated taxes on foods linked to obesity in general and also included simulation studies (e.g. Eyles 2012; Maniadakis 2013; Thow 2014). However, a systematic review of empirical evidence on the effect of taxing fat is lacking, despite existing examples of taxes on fat or saturated fat.

This research is part of a set of Cochrane Reviews of different types of food taxes, which are being carried out by the same author group and share the same methodological approach. Our reviews focus on the effects of governmental taxation on (1) the fat content of processed or packaged food (this review), (2) sugar‐sweetened beverages (Heise 2016), and (3) unprocessed sugar or sugar‐added foods (Pfinder 2016; Pfinder 2020).

## Objectives

To assess the effects of taxation of the fat content in food on consumption of total fat and saturated fat, energy intake, overweight, obesity, and other adverse health outcomes in the general population.

## Methods

### Criteria for considering studies for this review

#### Types of studies

Relevant evidence for this review was comprised of non‐RCT designs. This was expected, since the evaluation of real‐world taxation interventions is unlikely to be investigated in individual or cluster‐randomized studies (Lhachimi 2016b). Similarly, blinding is almost impossible in the evaluation of national‐level interventions. Therefore, and in order to summarize the ‘best available evidence’, we adapted an approach previously used in at least two other Cochrane Reviews, which considers evidence from various sources of the study designs (Gruen 2004; Turley 2013). This approach clearly separates studies into two broad categories: (1) studies meeting rigorous Cochrane EPOC (EPOC 2009; EPOC 2013) criteria, and (2) supporting studies, which do not meet EPOC criteria, and usually have a high risk of bias. According to EPOC, controlled before‐after studies require more than one intervention or control site, and interrupted time series studies require a clearly‐defined intervention time and at least three data points before, and three data points after, the intervention (EPOC 2013).

For the synthesis of the main results we included studies meeting the following Cochrane EPOC criteria:

- randomized controlled trials (RCTs);
- cluster‐randomized controlled trials (cRCTs);
- non‐randomized controlled trials (nRCTs);
- controlled before‐after (CBA) studies; and
- interrupted time series (ITS) studies.

There was no restriction in terms of publication date, publication status, language of publication (CPH 2011), or study duration.

##### Supporting studies

In accordance with our published protocol, we included as supporting studies (Lhachimi 2016b):

- studies using an RCT, cRCT, nRCT, CBA, or ITS design but not fulfilling the EPOC criteria;
- prospective cohort studies;
- retrospective/non‐concurrent cohort studies;
- repeated cross‐sectional studies; and
- uncontrolled before‐after (UBA) studies.

It was important to include supporting studies, since these may either support or challenge the results in the main findings. Also, supporting studies may highlight uncertainty and potential research gaps.

We excluded simulation studies due to the potential limitations introduced by their basic assumptions (e.g. lack of potential supply‐side changes, static models to predict weight loss), and other methodological considerations (e.g. the use of a combination of heterogeneous data sources) (Lin 2011; Shemilt 2015).

#### Types of participants

We included studies investigating participants of any age (children: zero to 17 years, and adults: 18 years and over), of any gender and from any country and setting.

We excluded studies that focused on specific subgroups only, particularly those fulfilling the following criteria at baseline and at the post‐intervention phase, due to their higher or lower health risks compared to the general population:

- people receiving pharmaceutical intervention;
- people undergoing a surgical intervention;
- pregnant females;
- professional athletes;
- ill people who are overweight or obese as a side effect, such as those with thyroiditis and depression; and
- people with chronic illness.

For these subgroups, the causal pathway of the effect of a tax on the fat content may differ from the general population.

#### Types of interventions

This review included studies that evaluated the effects of taxes on the fat content in foods. Such a tax can be expressed as sales, excise, or special VAT on the final product or an intermediary product (Chriqui 2008; Chriqui 2013; Jou 2012; Mytton 2012). Taxation may be calculated either as a share of the food’s weight, or as a share of the food’s energy. Since the taxation of fat is designed to incentivize the reduction in the amount of total or saturated fat in the production of a food item, or at least to incentivize consumers' replacement of saturated fat with other types of fat, we included studies evaluating the effect of fat taxation in both imported and domestically‐produced food items. The tax must have been applied both for imports and domestically‐produced food items.

We excluded virtual and hypothetical interventions imitating a taxation on the fat content in foods if participants’ purchase decisions are not binding so that they do not all result in a real purchase or if the money is virtual or not belonging to the study participant. We explicitly excluded import taxes that only target selected food items that are high in fat, as this is usually not being done to curb consumption of fats in general but to promote other domestically‐produced high‐fat products (e.g. butter).

We placed no restrictions on the duration of the intervention or whether taxation was applied at the local, regional, national, or multinational level. Also, studies evaluating the effects of artificial price increases of high‐saturated‐fat food that mimic taxation in clearly‐defined environments (e.g. cafeterias, supermarkets, and vending machines) were considered eligible (Epstein 2012). We included studies with any control intervention, such as no intervention, as well as other food taxes, bans, minimum pricing, media campaigns, or subsidies on healthy foods (Jou 2012; Thow 2011).

#### Types of outcome measures

Our outcome selection and grouping was guided by preliminary evidence, as discussed in the Background section, on the basis of the logic model (Figure 1), and feedback from the review advisory board members (see Table 2). Detailed information on advisory group involvement for this review is provided below. Primary outcomes also include intermediate health‐related outcomes directly affected by tax‐induced changes in food prices. That is, consumption and energy intake may directly alter the primary health outcomes of overweight and obesity. Secondary outcomes focused on food patterns (substitution and diet), expenditures, and other health outcomes directly or indirectly influenced by taxation of total fat/saturated fat content. We included demand, i.e. sales data, as a proxy for consumption (see How the intervention might work).

**1:** Advisory group members

**Name**	**Occupation**
Cristina Cleghorn	Department of Public Health, University of Otago, Wellington, NZ
Emilia Crighton	Faculty of Public Health, London, UK
Peter Faassen de Heer	Chief Medical Officer and Public Health Directorate, Scottish Government, Edinburgh, UK
Dionne Mackison	Department for International Development, UK Government, Glasgow, UK
Barry Popkin	Professor of Global Nutrition, University of North Carolina, Chapel Hill, US
Torben Jørgensen	Professor Department of Public Health University of Copenhagen, Copenhagen, DK

##### Primary outcomes

The review included changes from baseline to post‐intervention for the following primary outcomes.

##### Consumption

- Total fat consumption (e.g. frequency, amount)
- Consumption of saturated fat (e.g. frequency, amount)

##### Energy intake

- Total energy intake through fat
- Energy intake through saturated fat
- Total energy intake

##### Overweight and obesity

- Incidence of overweight and obesity
- Prevalence of overweight and obesity

All primary outcomes could be measured by physicians and other professionals, or self‐reported. Overweight and obesity can be measured by different anthropometric body mass indices, e.g. body weight, BMI, skinfold thickness, waist circumference (WC), waist‐to‐hip ratio (WHR), and waist‐to‐height ratio (WHtR), bioelectrical impedance analysis (BIA), magnetic resonance imaging (MRI), isotope dilution analysis (IDA), ultrasound, and computed tomography (CT) (WHO 2000). We planned to report changes in body mass indices if no data were available on the incidence or prevalence of overweight and obesity.

##### Secondary outcomes

The review included changes from baseline to post‐intervention for the following secondary outcomes.

##### Substitution and diet

- Composition of diet (expressed as food groups or ingredients, e.g. sugar, salt, fats)

##### Expenditures

- Total expenditures on food
- Total expenditures on processed or packaged food containing fat or saturated fat

##### Demand

- Total sales of processed or packaged food containing fat or saturated fat

##### Other health outcomes

- Health‐related quality of life (e.g. Short Form 36 (SF‐36), Health‐Related Quality of Life (HRQOL‐14))
- Mortality
- Any other health outcomes (e.g. type 2 diabetes, cardiovascular diseases)

### Search methods for identification of studies

We conducted various searches in order to find all relevant evidence for this review. We included systematic searches in electronic databases, searching for grey literature, internet searching, and we also undertook handsearching of reference lists of included studies.

#### Electronic searches

The search strategy was primarily developed for MEDLINE via OvidSP, and adapted to the other electronic databases. Our MEDLINE search strategy is documented in Appendix 1. The adapted search strategy for other electronic databases is documented in Appendix 2. Our search strategy was constructed using free‐text and controlled vocabulary. In order to increase the sensitivity of our search strategy, we did not apply filters for study types (Higgins 2019), or any other restrictions on publication date or publication format. The initial search in all electronic databases was conducted on 27 April 2016. We updated our search of all included electronic databases on 6 December 2016, 12 January 2018, and 12 September 2019. In total, 12 academic databases were searched:

- Campbell Library, via the Campbell Collaboration (2004 to 9 October 2019);
- Cochrane Central Register of Controlled Trials (CENTRAL; 2019, Issue 10) via Wiley (searched 9 October 2019);
- Cochrane Database of Systematic Reviews (CDSR), via Wiley (1995 to 9 October 2019);
- Cumulative Index to Nursing and Allied Health Literature (CINAHL), via EBSCO (1937 to 12 September 2019);
- Current Contents Medicine Database of German and German‐Language Journals (CCMed), via LIVIVO (1917 to 14 October 2019);
- EconLit, via EBSCO (1969 to 9 October 2019);
- Excerpta Medica database (Embase), via OvidSP (1947 to 12 September 2019);
- Food Science and Technology Abstracts (FSTA), via OvidSP (1969 to 14 October 2019);
- Latin American and Caribbean Health Sciences (LILACS), via BIREME/VHL (1982 to 12 September 2019);
- MEDLINE, via OvidSP (1946 to 12 September 2019);
- PsycINFO, via OvidSP (1887 to 9 October 2019); and
- Web of Science (SCI‐EXPANDED, SSCI, A&HCI, CPCI‐S, CPCI‐SSH, ESCI, CCR‐EXPANDED, IC), via Clarivate Analytics (1900 to 12 September 2019).

#### Grey literature databases

Our search strategy for grey literature databases is documented in Appendix 3. In total, we searched six databases, with the last update in October 2019:

- EconPapers, via REPEC (1997 to 14 October 2019);
- National Bureau of Economic Research (NBER) (1920 to 13 October 2019);
- ProQuest Dissertations & Theses Database (PQDT): UK and Ireland, via ProQuest (1637 to 9 October 2019);
- Social Science Research Network – SSRN eLibrary, via SSRN (1994 to 14 October 2019);
- System for Information on Grey Literature in Europe – OpenGrey, via OpenGrey (1994 to 9 October 2019); and
- The Directory of Open Access Repositories – OpenDOAR, via OpenDOAR (1739 to 12 December 2016; this database was not accessible in subsequent update searches).

#### Searching in clinical trial registries

Additionally, we searched for planned, ongoing, and completed (but not yet published) studies in two databases, using sensitive keywords relevant to the intervention (e.g. tax, taxation, pricing, etc.):

- Trials Register of Promoting Health Interventions (TRoPHI), via EPPI‐Centre (2004 to 11 August 2016; the free‐text search function was not accessible in subsequent update searches); and
- WHO International Clinical Trials Registry Platform (WHO ICTRP) (includes references of the ClinicalTrials.gov database), via WHO (1988 to 14 October 2019).

#### Internet search

We used the search engine Google Scholar and we also searched web pages of key organizations and institutions. The search strategy used in Google Scholar is documented in Appendix 4. Searches were conducted on 11 August 2016, and on 14 October 2019. The first 30 hits were screened.

The websites of the following key organizations and institutions were searched on 11 October 11 2019:

- Centers for Disease Control and Prevention (www.cdc.gov);
- DG Sanco (ec.europa.eu/dgs/health_food-safety/index_en.htm);
- European Commission (ec.europa.eu/index_en.htm);
- National Institute for Health and Care Excellence (www.nice.org.uk);
- Organisation for Economic Co‐operation and Development (www.oecd.org);
- WHO (www.who.int);
- World Cancer Research Fund Institute (www.wcrf.org);
- World Obesity Federation (www.worldobesity.org); and
- World Trade Organization (www.wto.org).

#### Searching other resources

We handsearched the reference lists of all included studies. We also asked our advisory group members to inform us of new or ongoing studies (see below for details). The last enquiry was on 26 February 2020 (Heise 2020).

#### Advisory group

We established a review advisory group of experts in the field of food taxation and health to comment and provide advice and suggestions to define important aspects along the review process. The review advisory group consisted of policymakers, researchers and academics. All members of the advisory group are documented in Table 2.

Experts from the advisory group were active during the protocol stage and gave advice on the definition of the specific research question (including relevance of the topic, study design, intervention, selected outcomes, search strategy and relevant databases, etc.). Experts were also involved during the development of the review, and during the preparation of this manuscript. Feedback from the advisory group members was collected via email and an online survey.

Following the GRADE approach, the advisory group members participated in an online survey and ranked pre‐selected outcomes according to their relative importance on a nine‐point Likert scale with the following categories: one to three: of limited importance; four to six: important; seven to nine: critical) (GRADE 2013). The results are documented in Table 3.

**2:** Feedback from advisory group

**1.1. Rank outcomes according to their relative importance for the scope of the reviews and general public health decision‐making in the context of food taxation;** 9‐point Likert scale (categories: 1 to 3 = of limited importance; 4 to 6 = important; 7 to 9 = critical)		
**Outcomes:**	**Average score:**	**Rank:**
Prevalence of overweight	7.67	3
Prevalence of obesity	7.67	3
Incidence of overweight	8.00	1
Incidence of obesity	8.00	1
Caloric intake through SSBs or unprocessed sugar/sugar‐added foods	7.33	8
Total calorie consumption	6.67	11
Consumption of sugar‐sweetened beverages (SSBs) or unprocessed sugar/sugar‐added foods (e.g. frequency, amount)	7.33	8
Health‐related quality of life	4.00	16
Total sales of SSBs or unprocessed sugar/sugar‐added foods	5.33	15
Composition of diet (e.g. fat, sugar, salt)	6.67	11
Total expenditures on food	4.00	16
Total expenditures on SSBs or unprocessed sugar/sugar‐added foods (e.g. frequency, amount)	5.67	14
Any health outcomes or health‐related unintended consequences	7.67	3
‐ e.g. mortality	7.00	10
‐ e.g. dental caries	6.00	13
‐ e.g. diabetes	7.67	3
‐ e.g. cardiovascular disease	7.67	3
**2.1. How well do the presented outcomes cover the basic review scope?**		
**Answers:**	**Rating:**	**Number of responses:**
Important outcomes are presented	66.67%	2
Important outcomes are missing	33.33%	1
Comments:	I imagine some evidence will be presented as simply a change in BMI or other markers of obesity rather than a change in incidence or prevalence of obesity (Cristina Cleghorn).	
**3.1. Do you think the same outcomes are appropriate for both reviews (SSB; sugar or sugar‐added foods)?**		
**Answers:**	**Rating:**	**Number of responses:**
The same group of outcomes should be utilized in both reviews	66.67%	2
Different outcomes should be utilised in the two reviews	33.33%	1
Comments:	Foods study: hard to go beyond kcal and weight and minimal cardio metabolic outcomes as the Morenga et al. review shows (Barry Popkin).	

### Data collection and analysis

#### Selection of studies

An information specialist (CF) and an additional review author (TLH) conducted the electronic database searches. One author (MB, TLH, or SKL) searched for grey literature, studies in the clinical trials registries, and conducted the internet searches. The screening process was done using the web‐based software Covidence (Covidence; Rathbone 2015). First, titles and abstracts (when available) were screened by at least two review authors (MB, TLH, SKL, UG, GG, FP, IS, or SVK prior to 2018; MB, TLH, or SKL in 2018 and 2019), independently from each other, considering pre‐defined eligibility criteria (see Criteria for considering studies for this review). At this stage, only obviously irrelevant articles were excluded. If an abstract was not provided by the database it originated from, and the title appeared to be potentially relevant, we progressed the record to full‐text screening. We resolved any disagreement by discussion and in consultation with a third author (SKL, TLH or MB), and eliminated all records that did not fit the inclusion criteria (see Criteria for considering studies for this review). We then retrieved the full text of potentially relevant records. These were screened by two review authors (FP and SKL), independently from each other, who documented reasons for excluding irrelevant articles. Both authors created a list with records that were considered to fulfil the inclusion criteria; they compared these lists and, in cases of disagreement, a third review author (TLH) made the final decision. At each stage we recorded the number of records retrieved and excluded in order to create the PRISMA flow chart (Liberati 2009). If a reference, abstract or full‐text report was in a language other than English, German or French, translation was performed by internet‐based translation tools or by native speakers.

#### Data extraction and management

We used reference management software (Endnote 2012) to store all records obtained by the electronic searches. Moreover, we used this software to administer the results of abstract and full‐text screening. At least two review authors (FP, TLH, and SKL) extracted data from the included full texts, while a third author resolved disagreements (SKL or TLH). For this process, we modified the data extraction and assessment template from Cochrane Public Health (CPH) (CPH 2011) for the complex intervention addressed in this review. Prior to the main data extraction process, MB, TLH, SVK, UG, FP, and SKL piloted and adapted the data extraction form to ensure standardized extraction. In accordance with our protocol (Lhachimi 2016b), data extraction and assessment included general information (publication type, country of study, funding source of study, potential conflict of interest), study eligibility (type of study, participants, type of intervention, duration of intervention, and type of outcome measures), study details (study aim, methods, results, intervention group, confounders, and confounder‐adjusted and unadjusted outcomes), indicators of changes in food prices, and other relevant information. Moreover, we extracted contextual factors that facilitate or hinder the implementation of the taxation on fat contents of foods, where available (e.g. political system, co‐interventions, reason for implementation, reason for particular tax level, intended beneficiaries, implementation costs, country and region‐specific level of gross domestic product (GDP), food security (availability, access, and use), and process evaluation criteria (e.g. satisfaction of participants, adherence) (Anderson 2011; Campbell 2018). We also used the PROGRESS categories (place of residence, race/ethnicity/culture/language, occupation, gender/sex, religion, education, socioeconomic status, social capital) to evaluate impacts on equity (O'Neill 2014).

References from all included studies were in English, thus no translation from other languages was necessary. As defined in our protocol, we contacted the authors of included studies to request additional data and information not reported in the identified publications.

#### Assessment of risk of bias in included studies

The risk of bias was evaluated for each included study independently by two review authors (FP, TLH, and SKL), with a third author (TLH or SKL) resolving disagreements. In accordance with our protocol (Lhachimi 2016b), risk of bias was assessed using different tools, depending on the nature of the study design. For the studies included in the main evidence synthesis (i.e. ITS studies), we assessed the risk of bias using the Cochrane ‘Risk of bias’ tool (Higgins 2011a), and the EPOC Group’s guidance (EPOC 2013). Both tools examine the following domains: selection bias, performance bias, detection bias, attrition bias, reporting bias, and other sources of bias. The EPOC 'Risk of bias' tool for ITS examines three further risks of bias: "was the intervention independent of other changes?", "was the shape of the intervention effect pre‐specified?" and "was the intervention unlikely to affect data collection?" (EPOC 2013). Each study was classified as having a low, high, or unclear risk of bias. For each judgement, supporting information was documented.

Risk of bias of 'supporting studies' (i.e. studies that did not meet EPOC criteria: cohort studies, repeated cross‐sectional studies, uncontrolled before‐after studies) was assessed with the Quality Assessment Tool for Quantitative Studies, developed by the Effective Public Health Practice Project (EPHPP 2010). This tool examines the following domains: selection bias, study design, confounders, blinding, data collection methods, withdrawals and dropouts, intervention integrity, analysis, and a global rating. As a result, each study is judged as having strong, moderate, or weak evidence (EPHPP 2010).

Studies were assessed at the level of the whole study, as all outcomes were considered to be comparable in risk of bias in this review. Following assessment of each domain, the overall risk of bias of a study was considered equal to the highest level of risk assessed for an individual domain. For example, if at least one domain was assessed as being at unclear risk, the study as a whole was considered at unclear risk.

#### Measures of treatment effect

For interrupted time series (ITS) studies, we reported the effect estimates as reported in each study. We confirmed that each ITS study had been analysed in an appropriate manner, including at least three time points before and after the intervention; a clearly identified intervention point; accounting for a possible time trend and possible seasonal effects; and accounting for possible autocorrelation (EPOC 2009; EPOC 2013; Polus 2017).

We did not identify more than one study per outcome measure, therefore we were not able to conduct a meta‐analysis. We intended to report the effects of the treatment on dichotomous outcomes as odds ratios (ORs), risk ratios (RRs) or risk differences (RDs) (Lhachimi 2016b). RRs are the preferred reported measure of treatment effect (CPH 2011). If RRs were not presented in a study, but data to calculate the RRs were provided, we planned to calculate them. This would have also applied for data suitable to calculate ORs (e.g. obesity prevalence). If data to calculate the RRs were not provided, we planned to contact the corresponding author of the study by email or phone to request the RRs or the data to calculate them. If we could not obtain RRs, we planned to report the treatment effect from the study report.

We planned to express continuous data as mean differences (MDs), where applicable, or as standardized mean differences (SMDs). Shorter ordinal data would have been translated into dichotomous data (expressed as ORs, RRs, or RDs) and longer ordinal data would have been treated as continuous data (expressed as MDs or SMDs). It is unclear whether there is a cut‐off point which is common across the studies and can be used for dichotomization (Higgins 2011a). The cut‐off point would have been part of a sensitivity analysis. We would have expressed count data and Poisson data as rate ratios. Time‐to‐event data (survival data) would have been translated into dichotomous data when appropriate, or into hazard ratios.

If feasible, we would have reported the adjusted treatment effect. If a study did not present adjusted treatment effect measures, we would have attempted to adjust the treatment effect measures for baseline variables by conducting additional multivariate analyses as far as we had access to the data, or by contacting the corresponding author of the study by email or phone to request the adjusted treatment effect measures. If studies presented intention‐to‐treat effect estimates, then we would have prioritized these over average causal treatment effect estimates (Higgins 2011a).

When the treatment effect had been described in cost estimates as derived from economic studies, we would have converted the cost estimates to US dollars (USD), and the price year to 2015, to compare cost estimates from different studies with each other. To convert cost estimates into USD, we would have applied an international exchange rate based on purchasing power parities. To convert cost estimates to the year 2015, we would have applied GDP deflators or implicit price deflators for GDP. Purchasing power parity conversion rates and GDP deflator values would have been derived from the International Monetary Fund in the World Economic Outlook database (www.imf.org/en/Data) (Higgins 2011a).

#### Unit of analysis issues

In the published protocol we had planned to consider the unit of analysis depending on study design (Lhachimi 2016b); in particular, we would have considered issues such as accounting for the effects of clustering or the level of allocation to an intervention/control group (i.e. individual or group). Since the included studies were ITS and one UBA study as supporting study, which do not have a control group, the unit of analysis was the study population included in the study (either supermarket‐level sales data or household‐level purchase data).

#### Dealing with missing data

We requested all missing information and data from study authors by email (Lhachimi 2020; Lhachimi 2020a; Lhachimi 2020b). We asked, in particular, for details on the study design, sample size, and (additional) measures of statistical precision for all included studies (see Characteristics of included studies).

According to our published protocol (Lhachimi 2016b) we intended to request all missing information and data from principal study authors by email or phone. In future updates of the review, according to our published protocol (Lhachimi 2016b), the following steps are to be taken to deal with relevant missing data:

- contact the authors;
- screen the study and investigate important numerical data such as randomized individuals as well as intention‐to‐treat, as‐treated, and per‐protocol populations;
- investigate attrition rates as part of the 'Risk of bias' assessment in terms of dropouts, losses to follow‐up and withdrawals;
- critically appraise issues of missing data and imputation methods (e.g. last observation carried forward);
- impute missing standard deviations if the authors contacted do not respond (Higgins 2011a); and
- apply sensitivity analyses to estimate the impact of imputation on meta‐analyses.

Data 'not missing at random' due to systematic loss to follow‐up or systematic exclusion of individuals from studies would have been sought and requested from study authors (Higgins 2011a).

#### Assessment of heterogeneity

We planned to perform meta‐analysis only where there was no substantial heterogeneity between included studies for a specific outcome (Lhachimi 2016b). Due to the low number of included studies, we did not perform a meta‐analysis and therefore inspection of statistical heterogeneity was not possible. Nevertheless, we narratively described the methodological heterogeneity of the included studies, considering study population, intervention characteristics, implementation level, and outcomes.

#### Assessment of reporting biases

Reporting biases — including publication bias, time‐lag bias, multiple (duplicate) publication bias, location bias, citation bias, language bias, and outcome reporting bias — occur when the dissemination of research results depend on their magnitude and direction (Higgins 2011a). We planned to inspect reporting bias with funnel plots in the case that we had at least ten studies investigating the same outcome (Lhachimi 2016b). Since this was not the case, we were not able to analyze reporting bias with funnel plots.

#### Data synthesis

If two or more studies reported the same outcome and were sufficiently homogenous conceptually, methodologically, and statistically, we planned to perform meta‐analyses of these outcomes using Review Manager 5 (Review Manager 2014). Since insufficient studies were included to perform meta‐analysis, results were described narratively. To conduct narrative synthesis, we considered direction of effect as our common metric across studies to establish whether there is evidence of an effect of taxation in the available literature. We grouped individual studies by outcome categories, tabulated key information from each study, and summarized the pattern of findings according to outcome (Campbell 2020).

#### Subgroup analysis and investigation of heterogeneity

The included studies did not provide sufficient data to conduct subgroup analysis. In the published protocol (Lhachimi 2016b), we had planned to investigate the following subgroups for the primary outcomes:

- high‐income countries versus low‐ and middle‐income countries;
- high‐income groups versus middle‐income groups;
- single tax versus multiple taxes on fat content;
- tax on saturated fat alone versus tax on saturated fat accompanied by other fat taxes;
- tax on fat accompanied by other interventions (e.g. bans, minimum pricing, media campaigns, or subsidies of healthy foods);
- different types of taxation (e.g. excise tax or VAT);
- children versus adults; and
- BMI.

#### Sensitivity analysis

We had planned to conduct sensitivity analyses by removing studies with a high risk of bias and by removing outliers contributing to statistical heterogeneity (e.g. different study designs, sources of study funding, different study follow‐up times). However, not enough studies were included in the review to conduct sensitivity analysis.

#### Summary of findings and assessment of the certainty of the evidence

We generated a ‘Summary of findings’ table containing the outcomes reported across the included studies. Additionally, in accordance with our protocol (Lhachimi 2016b), we included a ‘Summary of findings’ table for outcomes reported across supporting studies. Based on the feedback provided by our advisory board and external reviewers, we considered including at least the following pre‐selected outcomes: total fat consumption, consumption of saturated fat, total energy intake, composition of diet prevalence of overweight or obesity, and total sales. 'Summary of findings' tables include information on the outcomes, results provided by the study, the sample size, the number of studies included, the quality of evidence based on GRADE (Schünemann 2013), and additional comments. The assessment was done by two review authors (TLH and SKL). We used GRADEprofiler software to prepare the ‘Summary of findings’ table (GRADE 2013; GRADEpro GDT; Higgins 2011a).

Within the GRADE approach, the certainty of evidence is assessed based on a number of factors which affect the certainty of the evidence. There are four possible levels of certainty (observational studies with an ITS design begin with the level 'low certainty'):

- high‐certainty (further research is very unlikely to change our confidence in the estimate of effect);
- moderate‐certainty (further research is likely to have an important impact on our confidence in the estimate of effect and may change the estimate);
- low‐certainty (further research is very likely to have an important impact on our confidence in the estimate of effect and is likely to change the estimate); and
- very low‐certainty (any estimate of effect is uncertain).

There are five factors that for which the certainty of evidence can be downgraded:

- risk of bias of individual studies (limitations in the design and implementation of available studies suggesting high likelihood of bias);
- indirectness of evidence (indirect population, intervention, control, outcomes);
- unexplained heterogeneity or inconsistency of results;
- imprecision of results; and
- high probability of publication bias.

There are three factors for which the certainty of evidence can be upgraded:

- large magnitude of effect;
- all plausible confounding would reduce a demonstrated effect or suggest a spurious effect when results show no effect; and
- dose‐response gradient.

## Results

### Description of studies

See Characteristics of included studies and Characteristics of excluded studies.

#### Results of the search

We identified 23,281 records from searching electronic databases and 1173 records from other sources (including grey literature databases with 802 records), leading to a total of 24,454 records. After removal of duplicates 18,767 records were included in the abstract screening using Covidence. In total, we studied eight articles at the full‐text stage. Of these, five were excluded: four considered a different intervention (Elbel 2013; Hannan 2002; Khan 2015; Taillie 2017) and one was a modelling study (Smed 2016). This resulted in two studies being included in the analysis (Jensen 2013; Jensen 2015), and one supporting study (Bodker 2015 (supporting study)). We documented the results of the study selection process in a PRISMA flow diagram (Figure 2).

**2:**
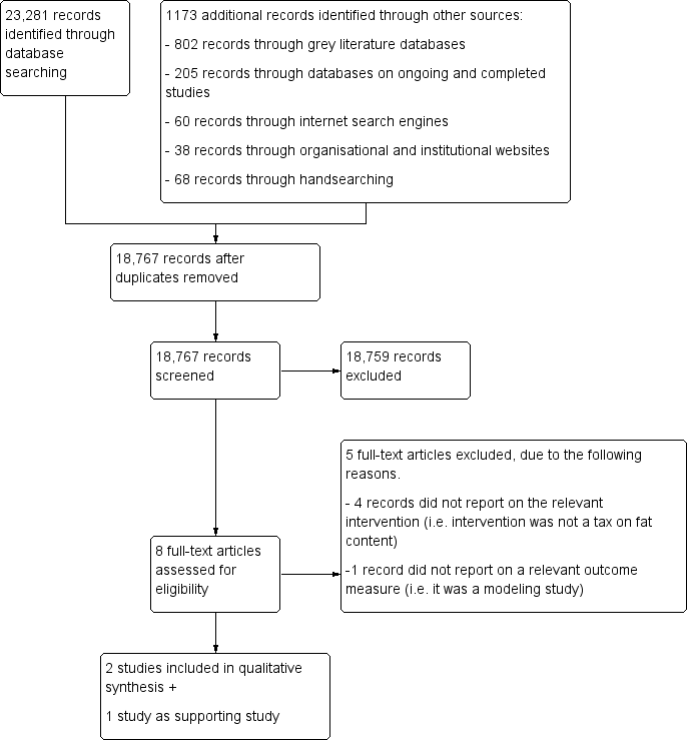
Study flow diagram.

We conducted our searches in intervals, with the last search taking place in September 2019 (see Electronic searches for details).

#### Included studies

We included two studies (Jensen 2013; Jensen 2015), both of which investigated the effect of the Danish tax on the content of saturated fat in selected food items.

##### Supporting studies

We included one study as a supporting study, which also investigated the Danish tax (Bodker 2015 (supporting study)).

##### Study design and participants

Both included studies were retrospective ITS, fully compliant with the EPOC criteria (EPOC 2009; EPOC 2013).

The first study (Jensen 2013) investigated the effect of the Danish fat tax on demand for selected food products that are high in fat content, i.e. butter, blends, margarine, and oil. The study population was a panel of approximately 2000 Danish households from 1 January 2009 to 1 July 2012. The panel was unbalanced because about 20% of all households were replaced each year by similar types of households. The participating households recorded all their purchases by price and quantity. For the analysis, the household purchases were aggregated to report weekly purchases. The statistical model specification to estimate the effect of the intervention was a Tobit model, to account for households that had zero consumption; it was also adjusted for household characteristics and seasonal effects. This study fulfills the criteria as outlined by the EPOC guidance to be included as an ITS study, i.e. at least three time points before and after the intervention; a clearly identified intervention point; accounting for a possible time trend and possible seasonal effects; and accounting for possible autocorrelation (EPOC 2009; EPOC 2013). Hence, the employed study design is considered highly appropriate for ITS studies (Polus 2017).

The second study (Jensen 2015) investigated the effect of the Danish fat tax on demand for selected food products that are potentially high in fat content, i.e. minced beef, regular cream, and sour cream. The study populations were shoppers of a particular Danish supermarket chain with a market share of approximately 40%. The authors analyzed the monthly sales volume and sales revenue recorded by a balanced panel of 1,293 supermarkets (i.e. the same supermarkets throughout the time period). For each included food product the fat content was on record (National Food Institute 2009). The statistical model specification to estimate the effect of the intervention on sales was a fixed‐effect regression which accounted for seasonal effects, time‐trends and shifts in overall demand, in addition to the effect of the intervention itself. This study fulfills the criteria as outlined by the EPOC guidance (EPOC 2009; EPOC 2013) to be included as an ITS study: i.e. at least three time points before and after the intervention; a clearly identified intervention point; accounting for a possible time trend and possible seasonal effects; and accounting for possible autocorrelation. Hence, the employed study design is considered highly appropriate for ITS studies (Polus 2017).

###### Supporting studies

The supporting study (Bodker 2015 (supporting study)) was an UBA that did not have a sufficient number of observed time points before and after the intervention to fulfil the EPOC criteria for an ITS study. The main objective of the study was to project the health effects of changes in consumption of saturated fat using a simulation tool. The analysis of sales data was merely an input into the simulation tool and change in population‐level risk for ischemic heart disease was the simulation output. The study population was shoppers from all Danish outlet chains (except two discounts chains); the number of supermarkets or observations in the analysis were not reported. The data covered the total sales of 12 food products high in fat content, i.e. butter, butter blends, margarine, fat, oil, cheese, cream, sour cream, chips, snacks, cookies, and biscuits. For all food products the fat content was calculated. The sales data were collected for the first 28 weeks of each year under observation (2010 to 2013) and aggregated into a single time point for each year.

##### Intervention

The two studies included in this review (Jensen 2013; Jensen 2015) investigated the effect of the Danish tax on saturated fat. The tax came into effect on 1 October 2011, and was subsequently repealed by an act of parliament in November 2012. Hence, the tax was still implemented until 31 December 2012. The tax covered only certain food types, including meat, full‐fat dairy products, animal fats, edible oils, and margarine; it exempted food items with a saturated fat content of 2.3% or less. The tax was designed as an excise tax and the rate was set at 16 Danish krone (DKK) (approximately USD 2.90 in 2012) per kilogram of saturated fat contained in the food item, plus 25% VAT (see Jensen 2015 for more details).

Jensen 2013 covered a pre‐intervention period from 1 January 2009 to 30 September 2011 (196 weeks); the actual intervention period started on 1 October 2011 and lasted for 39 weeks until the end of the study. The authors accounted for a potential hoarding effect by including a dummy variable for the two‐week period before the tax was implemented.

Jensen 2015 covered a pre‐intervention period from 1 January 2010 to 30 September 2011 (91 weeks); the actual intervention period started on 1 October 2011 for 57 weeks until the end of the study (31 October 2012). The authors accounted for a potential hoarding effect by including a dummy variable for the month September of 2011 (i.e. four weeks before implementation of the tax).

###### Supporting studies

Bodker 2015 (supporting study) also investigated the Danish tax, and covered a pre‐intervention period for 48 weeks in total: from January 2010 to July 2010 (28 weeks) and January 2011 to July 2011 (28 weeks). The actual intervention period was covered for 28 weeks from January 2012 to July 2012, and the post‐intervention period ranged from January 2013 to July 2013 (28 weeks). Therefore, the period directly before and after the implementation of the intervention was excluded.

##### Context and implementation

All included studies investigated the effect of a particular intervention, i.e. the Danish tax on saturated fat. Discussions in Denmark on a tax on saturated fat can be traced back to 2009. The underlying idea of proposing such a tax was to use differentiated pricing on food products to incentivize healthy eating habits and increase overall population health (CoP 2009). In August 2009, the first draft of the tax bill was introduced in parliament and the final version of the bill was discussed in parliament in January 2011; it finally passed in March 2011. The proposed tax rate was changed during the discussion of the bill from initially DKK 25 to DKK 13 and finally to DKK 16 for each kilogram of saturated fat. Additionally, a threshold of 2.3% saturated fat in the products was set, exempting all products with less saturated fat content, in particular regular drinking milk and milk‐based yoghurts.

Some scholars have argued that the main motivation of the Danish government for introducing the tax was primarily to raise additional revenue, not to improve population health (Bødker 2015a; Jensen 2018; Jørgensen 2016; Vallgarda 2015). For example, the tax was part of a larger package of financial bills and the final level of the tax on saturated fat content was determined in such a way to ensure a certain amount of revenue to offset the costs of simultaneously decreasing taxes on labour (Bødker 2015a). Moreover, the Danish government had no plans for monitoring the health consequences of the bill, although the revenue effects of the bill were to be monitored closely (Vallgarda 2015). Similarly, the main argument for the repeal was economic. In particular, the cost for companies and retailers in administering the tax and also job losses for food producers were put forward as the main arguments against the tax. Additionally, the tax received substantial negative media coverage. Already in November 2012, the parliament voted to repeal the tax, starting 1 January 2013. Hence, the tax was in effect for 15 months and no evaluation of the health effects of the bill was published during this period (Vallgarda 2015).

##### Outcome measures

###### Primary outcomes

Both studies reported estimates for the consumption of at least one primary outcome measure: Jensen 2013 reported on the total fat consumption, and Jensen 2015 on the saturated fat consumption. Both studies, however, used the changes in sales/purchases of food products (collected at store or household‐level) as a proxy to estimate from those changes the average change at the individual level. No study recorded consumption or intake at the individual level. None of the included studies reported on the incidence of overweight or obesity.

Jensen 2013 included only four different types of food products that are rich in fats and saturated fat, i.e. butter, blends, margarine, and oil. The household purchases of these four food products were measured as grams per week, summed up and divided by the number of individuals in the household, in order to estimate the total fat consumption per person. Possible variations in the actual level of fat content of the different products were not accounted for. Moreover, no estimate was given about the level of saturated fat content.

Jensen 2015 included only three different types of food products (i.e. minced beef, regular cream, and sour cream), subdivided into products with low fat content (less than 7% fat content), medium fat content (7% to 11% fat content), and high fat content (more than 11% fat content). According to the study authors, these three types of food products jointly represent an estimated 10% to 15% of Danes’ total intake of saturated fat. The average change in sales of these food products was estimated using the pooled supermarket sales data. The saturated fat content of total sales was calculated using product‐specific coefficients for saturated fat content using the Danish Food Composition Database (National Food Institute 2009). For their estimate of the average percentage change in saturated fat consumption, based on changes of total sales of all three products, the authors did not report confidence intervals, significance levels (i.e. P values), or any other measure about the statistical precision of their effect estimates.

##### Secondary outcomes

###### Substitution and diet

Jensen 2015 reported the changes in the distribution of sales as a consequence of the tax for all three included food products, i.e. from a high‐fat variety to a medium‐ or low‐fat variety, based on supermarket sales data. The authors, however, did not report confidence intervals, significance levels (i.e. P values), or any other measure about the statistical precision of their effect estimates.

####### Supporting studies

Bodker 2015 (supporting study) reported the total sales of all food products under investigation in metric tonnes. They also reported percentage changes in sales for all included food products. However, they did not report confidence intervals, significance levels (i.e. P values), or any other measure about the statistical precision of their effect estimates.

##### Funding and conflict of interests

Jensen 2015 received funding from the Danish Ministry of Science, and the authors declared that they have no conflicts of interests. Jensen 2013 did not state any sources of funding and did not provide a statement about potential conflicts of interests.

###### Supporting studies

Bodker 2015 (supporting study) received funding from the Danish Health Foundation ('Helsefonden', a charity foundation to improve population health), and the authors declared that they have no conflicts of interest.

#### Excluded studies

We excluded five studies from our analysis. The study by Smed 2016 combined estimates for sales reduction (which were not reported) with heterogeneous data sources (e.g. additional survey data collected at different time points) and therefore constituted a modelling study. Two studies did not investigate a tax as an intervention (Hannan 2002; Khan 2015), and two further studies did not target to tax the fat content of food (Elbel 2013; Taillie 2017). Full details for exclusion are shown in Characteristics of excluded studies.

#### Studies awaiting classification

We did not identify any study awaiting classification.

#### Ongoing studies

We did not identify any ongoing studies.

### Risk of bias in included studies

Judgements from the 'Risk of bias' assessment are summarized under Characteristics of included studies. The included studies (both of which had an ITS design) were judged overall to have an unclear risk of bias (Jensen 2013; Jensen 2015). Figure 3 shows the 'Risk of bias' judgement for each domain of each included study and the supporting study.

**3:**
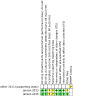
Risk of bias summary: review authors' judgements about each risk of bias item for each included study (blank cells indicate that the particular domain was not assessed for the study).

#### Allocation

Generation of allocation sequence and adequate concealment of allocation sequence is not applicable in ITS studies (according to the EPOC criteria).

#### Blinding

The intervention itself, a national tax, was not blinded. However, the participants in all studies were not aware that their data were used to investigate the effect of the tax. Hence, the risk of performance bias was judged to be low for both studies included.

#### Intervention independent of other changes

The intervention in all included studies was a legislative act that did not have health outcomes as a motivation and was implemented without any cointervention. Hence, we judged the risk of bias for this domain to be low for all studies.

#### Shape of effect pre‐specified

For all included studies, the shape of the effect of implementing a tax on saturated fat was pre‐specified, informed by micro‐economic theory before testing the intervention effect. Hence, we judged the risk of bias for this domain to be low for all included studies.

#### Intervention unlikely to affect data collection

For all included studies the analysis was done using databases that are permanent data collections for the purpose of market research (either household panels or through supermarket cashiers). The data collection was initiated several years before the intervention and was also continued several years after the data collection. Hence, we judged the risk of bias for this domain to be low for all included studies.

#### Incomplete outcome data

Jensen 2013 reported that their unbalanced panel had an annual attrition/replacement level of 20%. No further analysis was provided to what extent this attrition and replacement may affect outcome measures. Hence, we judged the risk of bias in this domain as unclear. Jensen 2015 did not report on the completeness of their outcome data, and so we judged the risk of bias in this domain as unclear.

#### Selective reporting

None of the studies were based on published study protocols, however, the outcomes described in the methods sections were reported. This is not unusual for ITS studies. The data were derived from databases that are permanent, commercial data collections for the purpose of market research potentially covering a wide range of products, yet both studies only included certain food types and did not provide a clear rationale for selecting particular food products. Hence, we judged the risk of bias for both studies as unclear.

#### Other potential sources of bias

The data in Jensen 2013 was based on consumer panels where households self‐record their shopping (prices and quantities). Such households might be more price‐sensitive than the general population, where individuals may or may not have the same level of awareness concerning changes in prices or their spending patterns (or both). We judged the risk of bias from this source as being unclear.

#### Risk of bias in supporting studies

The risk of bias of the included supporting study (Bodker 2015 (supporting study)) was assessed using the EPHPP criteria, and was judged as weak (i.e. the study was rated as having weak evidence).

### Effects of interventions

See: Table 1

#### Effects of taxation of the fat content of food for reducing their consumption and preventing obesity or other adverse health outcomes

The effects of taxation of the fat content of food were investigated in two included studies without any control group. As we did not identify more than one study per outcome measure, we were not able to conduct a meta‐analysis. Thus, we have summarized the results narratively in the following section.

A summary of the findings on the effects of the taxation of the fat content of food, based on the two included studies, is presented in Table 1. For an overview of the studies, see Table 4.

**3:** Overview of included studies and supporting studies by outcome

**Study**	**Study Design**	**Overall risk of Bias**	**Intervention**	**Measurement of outcome**	**Description of effect**
*Consumption of total fat*					
Jensen 2013	ITS (2000 households)	Unclear	Danish tax on saturated fat	Calculations of change in total fat are based on (1) selected food products (i.e. butter, blends, margarines, and oils), (2) using sales as proxy for consumption, and (3) assumptions about average consumption per person in a household	A decrease in total fat content consumption
*Consumption of saturated fat*					
Jensen 2015	ITS (1293 supermarkets)	Unclear	Danish tax on saturated fat	Calculations of change in saturated fats are based on (1) selected food products (i.e. minced beef, cream, sour cream), (2) using sales as proxy for consumption, and (3) using data from a particular supermarket chain covering 40% of market share	A decrease in saturated fat consumption from minced beef and cream, and an increase from sour cream
*Substitution*					
Jensen 2015	ITS (1293 supermarkets)	Unclear	Danish tax on saturated fat	Calculations to what extend a high‐fat variety of a food item is substituted by low‐fat variety of this food item based on (1) selected food products (i.e. minced beef, cream, sour cream), (2) using sales as proxy for consumption, and (3) using data from a particular supermarket chain covering 40% of market share	Substitution effect within one product category from the high variety to a low fat variety (e.g. more sales of low‐ and medium‐fat and less sales of high‐fat minced beef)
*Demand*					
Bodker 2015 (supporting study)	UBA (unclear number of included shoppers)	Weak	Danish tax on saturated fat	Change of total sales of selected food products in shoppers from Danish outlet chains measures, in metric tonnes per year.	Total sales of selected food products decreased in the year while the tax was implemented, while also showing that in the year before and after the tax was implemented, total sales increased.

ITS: interrupted time series UBA: uncontrolled before‐after

#### Primary outcomes

##### Consumption

###### Changes in total fat consumption

This outcome was reported in one of the included studies, which analyzed the effect of Danish tax on saturated fat for a selection of four food products: butter, blends, margarine, and oil (Jensen 2013). The study population consisted of approximately 2000 households (no individual participant data) and the results showed a mean reduction in total fat consumption of 41.8 grams per week per person in each household (P < 0.001; no precise P value was reported), based on the four food products investigated. Our calculations show this is equivalent to a reduction of approximately six grams per person per day. We judged the certainty of this evidence to be very low, according to GRADE criteria.

###### Changes in consumption of saturated of fat

One of the included studies (Jensen 2015) assessed changes in consumption of different types of fat. The study investigated sales data from 1293 Danish supermarkets, in order to estimate consumption of selected food products (minced beef, regular cream, and sour cream). The authors reported a mean percentage reduction of the saturated fat content of all minced beef sales of 4.2%, and a reduction of 5.8% of saturated fat content of all cream sales. However, they did find a slight increase of 0.5% for the saturated fat content of the total sales of sour cream. The study did not report absolute values for this outcome, and no measures of statistical precision were reported for any of these results. The direction of the effect is in line with expectations from economic theory, i.e. an increase in price leads to a decrease in consumption. However, we judged the certainty of the evidence for the total saturated fat consumption to be very low, according to GRADE criteria.

##### Energy intake

None of the included studies reported on total energy intake through fat, energy intake through saturated fat, or total energy intake.

##### Overweight and obesity

None of the included studies reported on the incidence or prevalence of overweight and obesity.

#### Secondary outcomes

##### Substitution and diet

Jensen 2015 reported estimates that showed substitution effects within one product category (e.g. low‐ and medium‐fat versus high‐fat minced beef), according to the sales data from 1293 Danish supermarkets. The demand for high‐fat minced meat decreased by 10.6%, while the demand for low‐fat minced meat increased by 5.1%. Similarly, demand for low‐fat sour cream increased by 4.6%, while the demand for the high‐fat variety decreased by 8.6%. No measures of statistical precision were reported for any of these results. The direction of this effect is in line with the expectation from economic theory.

##### Expenditures

None of the included studies reported on total expenditures on food or total expenditures on processed or packaged food containing fat or saturated fat.

##### Demand

None of the included studies reported on changes in overall demand. Nevertheless, a crucial element about the intervention investigated in this review is to know if (and to what extent) an excise tax is being passed on to the consumer, i.e. do producers and retailers raise prices on products (see Figure 1)? This was the case for the Danish tax on saturated fat as investigated by the two studies included in this review. Jensen 2013 reported data from approximately 2000 households, that discount stores passed on taxes in full to consumers for blends and oils; and for butter and margarine the pass‐on rate was even higher than the expected amount, that is, the retailers used the implementation of the tax to increase profit margins. For non‐discounting supermarkets, the pass‐on rate for the four product categories investigated was slightly lower than for discount supermarkets. Moreover, Jensen 2015 showed that prices for the high‐fat variety of minced beef, cream, and sour cream increased by 16% (P < 0.01; no precise P value was reported), 14% (P < 0.01; no precise P value was reported), and 13% (P < 0.01; no precise P value was reported), respectively (according to sales data from 1293 Danish supermarkets). The prices for the low‐fat variety of these products decreased slightly, by 1% for minced beef (not significant; no precise P value was reported), 2% for cream (P < 0.01; no precise P value was reported), and 1% for sour cream (P <0.01; no precise P value was reported).

###### Supporting studies

Bodker 2015 (supporting study) reported on the total sales of selected food products from Danish outlet chains (the number of supermarkets or observations in the analysis were not reported). The study showed that, for the food products under investigation, total sales decreased by 911 metric tonnes, a decrease of 0.9% (no measure of statistical precision was reported). They also found that the total sales increased by 1.3% from 2010 to 2011 (the time before the tax implementation). An increase of 1.3% (no measure of statistical precision was reported) was also observed from 2012 to 2013, which is the time after the tax was abolished.

##### Other health outcomes

None of the included studies reported on health‐related quality of life (e.g. Short Form 36 (SF‐36) or Health‐Related Quality of Life (HRQOL‐14)); mortality; or any other health outcomes (e.g. type 2 diabetes, cardiovascular diseases).

## Discussion

### Summary of main results

We aimed to assess the effects of taxation of fat content in food on consumption of total fat and saturated fat, energy intake, overweight, obesity, and other adverse health outcomes in the general population. In our search, we identified 23,281 records from searching electronic databases and 1173 records from other sources — in particular, grey literature databases, from which we retrieved 802 records — leading to a total of 24,454 records. We included two studies in the analysis, both of which were interrupted time series (ITS) studies; additionally, we analyzed one uncontrolled before‐after study as a supporting study. We judged the overall risk of bias of the two included studies as 'unclear', and the supporting study as 'weak'. We were not able to conduct a meta‐analysis because we did not identify more than one study per outcome measure.

For the primary outcome, total consumption of fat, a mean reduction of 41.8 grams per week per person in each household (P value < 0.001; no precise P value was reported) was identified. We calculated that this equates to a mean difference of approximately ‐6 grams per person, per day. Considering that the average total fat intake in Denmark is 111 grams per day (standard deviation: 39.1 grams per day) for males, and 83 grams per day (standard deviation: 28.8 grams per day) for females — i.e. 777 grams per week for males and 581 grams per week for females — we consider the reduction of 6 grams per day to be clinically meaningful (Nadelmann 2015). However, this estimate is based on a restricted number of food types and does not account for other sources of fat intake that were not included in the study. For example, packaged foods or foods consumed at other outlets (e.g. restaurants, cafeterias, etc.) were not included. Furthermore, the analysis did not account for heterogeneity in the fat consumption pattern of households or individuals, which vary in Denmark by age, sex, and other characteristics such as income and education (Rasmussen 2012). Hence, we judged the evidence about the effect of taxation of fat contents of food on consumption of total fat to be very uncertain. One study reported a reduction in the sales of selected food items high in saturated fat content, e.g. minced beef; similarly, we judged the evidence on the effect of taxation of fat contents of food on consumption of saturated fat to be very uncertain.

Comparing the identified evidence with our logical model, we are a very uncertain about the effect of the Danish tax. However, for a very limited number of food items that were investigated in the included study, we can identify that the Danish tax had a detectable influence on certain prices and consumer behaviour: supermarkets altered their prices and consumers altered their purchases (Figure 4 shows the adapted logical model). However, only a restricted number of food products were part of the analysis, and no statement on the overall effect on prices and consumer behaviour can be made. For example, cheese or other dairy such as ice cream were not included; also no packaged or pre‐processed foods were investigated. Moreover, other food outlets such as convenience stores, restaurants, or cafeterias were not covered by the studies.

**4:**
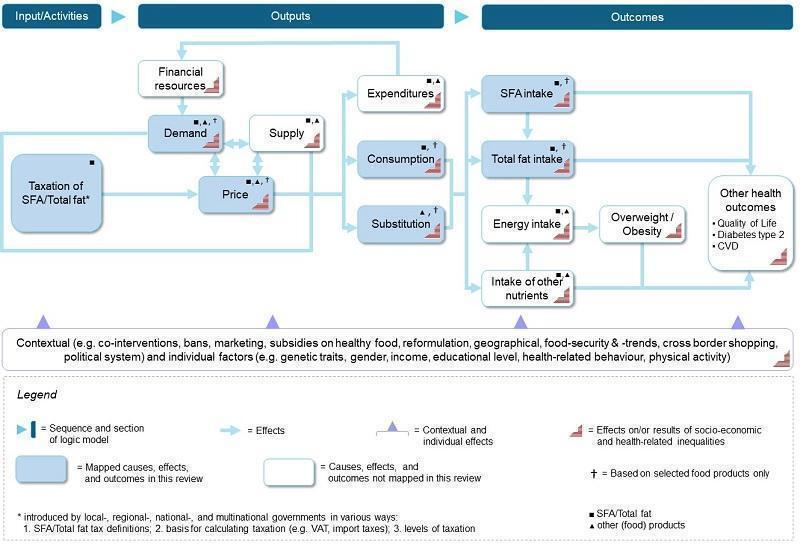
Adapted logical model

Due to the very low certainty of the evidence for the identified outcomes (total fat and saturated fat consumption), and the absence of evidence on other outcomes (i.e. total energy intake, incidence and prevalence of overweight or obesity), we cannot be certain about the direction and absolute effect of the taxation intervention on total fat or saturated fat consumption. Moreover, we cannot be certain about the direction and absolute effect of substituting taxed food products with other more or less harmful products or behaviours.

### Overall completeness and applicability of evidence

The body of evidence based on the identified studies is incomplete. We identified only one country which evaluated the effects of implementing a tax on fat contents on food. Primary outcome measures were either addressed insufficiently (consumption of fat) or not at all (energy intake, overweight and obesity). Moreover, all identified studies used sales data only (either recorded at the supermarket or the household level). Hence, there is no information on how individual diet and individual fat intake (as is often collected in nutrition surveys) changed due to the intervention. Sales data were also usually recorded either at store‐level or at household‐level, which represents a heterogeneous study population that cannot be accounted for (e.g. households may have children or not, etc.). Also, the sales data do not account for purchases in food outlets or for fat intake outside the household; and none of the studies considered the potential impact of cross‐border shopping (a practice to exploit taxation differentials, which has been well‐studied in Nordic countries (Asplund 2001)). The studies may not be representative of the Danish population, because they are based on convenience samples (supermarket‐level sales data or existing consumer panels of a private company). Moreover, the included study investigated only one particular intervention in Denmark only; this limits the applicability of the findings to other settings/countries, and other designs of the tax intervention.

We planned to include certain studies as supporting studies to increase the possible evidence base. Despite their methodological limitations, supporting studies may enable more insight on the intermediate steps, as depicted in our logical model, of the causal pathway of a taxation of the fat or saturated fat content of food. The supporting study we included (Bodker 2015 (supporting study)) also indicated that overall food demand changed when the Danish tax was implemented.

### Quality of the evidence

We assessed the certainty of evidence as very low for reducing consumption of total fat or saturated fat. We had to downgrade the findings of the primary outcomes due to indirectness: firstly, because the studies by Jensen 2013 and Jensen 2015 focused only on certain food products (although the databases used — self‐recorded household purchase and supermarket sales data — in principle may contain all sold food products); and secondly, because sales/purchases of food items was used as a proxy to measure consumption. Moreover, we had to downgrade the findings for total saturated fat consumption and substitution due to imprecision, since no measure of statistical precision was reported. We did not identify any evidence on the effect of the intervention on total energy intake, energy intake through saturated fat or total fat, or prevention of the incidence or reduction of the prevalence of overweight or obesity.

Several additional limitations must be acknowledged. First, the included studies conducted their analysis only with a limited number of food types. These studies cannot account fully for substitution effects of shoppers. Hence, we do not have information about the change in total fat consumption or saturated fat consumption. Second, these studies use already‐existing data sets that potentially cover a much larger range of food types that could be included in the analysis. There is no clear rationale as to why the included food types were selected and others not. This strongly indicates that analysis of already‐existing data should be guided by prespecified protocols. Finally, none of the included studies investigated any health‐related outcomes, including potential harms or adverse events.

### Potential biases in the review process

We are confident that we identified all present or past eligible interventions, i.e. taxation of the fat content of food products. As our review is part of a larger project concerning the taxation of food products to curb overweight/obesity and other adverse health outcomes (Heise 2016; Pfinder 2020), we believe that we would be aware of applicable interventions in other countries, especially considering the public nature of tax legislation and the relatively large administrative effort needed to implement such a tax in jurisdiction. Moreover, we contacted our advisory board members regularly to ask if they were aware of any ongoing studies (Heise 2020). For the only applicable case, the Danish tax on saturated fat, we are sufficiently confident that we identified all eligible published studies. However, it is unclear to what extent private companies conducted market research on the changes in sales of affected food products that remained unpublished.

We did not make any major changes during the review process compared with our published protocol (Lhachimi 2016b) (see Differences between protocol and review for further details). Hence, we judge the potential risk of bias in this review process as low.

### Agreements and disagreements with other studies or reviews

We are not aware of any other studies or systematic reviews about the effect of taxation of fat or saturated fat content in food.

## Authors' conclusions

**Implications for practice**

Due to the very low certainty of the available evidence, we are not able to conclude whether a tax on fat or saturated fat is effective or ineffective in reducing total consumption of fat or saturated fat. Moreover, we did not identify any evidence on whether a tax on fat or saturated fat is effective or ineffective at reducing total energy intake, the incidence or prevalence of overweight or obesity, or other adverse health outcomes.

**Implications for research**

High‐quality studies on the effect of a tax on fat or saturated fat are needed in order to understand its effectiveness in reducing the consumption of fat/saturated fat, and thereby preventing obesity or other adverse health outcomes. It is notable that we did not identify any study with a dedicated epidemiological research objective and more comprehensive research design, as is available for legislation on the availability of alcohol (Nelson 2017). The effectiveness of taxation cannot be studied using a standard RCT approach; instead, natural experimental studies are required (Craig 2017). Considering this, the Danish taxation on saturated fat from 2011 to 2012 is a missed opportunity for public health research, especially in light of the evidence we identified to suggest it had a detectable influence on certain prices and consumer behaviour. To our knowledge, no prospective research project was planned or initiated by the Danish authorities.

Even though the Danish fat tax is now abolished, more comprehensive research about its effects is, in principle, feasible. An advantage of ITS studies is that they can be used to retrospectively analyze data that have been already collected (perhaps originally for a different purpose). The existing data on household purchases should be investigated using a more comprehensive approach, that is, including all food types and not only a limited number. Nevertheless, reliable studies on nutrition intake are very demanding in terms of research design and should preferably always be prospective; and even a well‐conducted ITS study cannot establish the same level of certainty as a well‐designed and conducted RCT. Moreover, the Danish fat tax (and the oversights in terms of formally evaluating it) is a good example of the need to implement a health in all policies (HiaP) approach (Kickbusch 2013), a crucial element of which is to conduct health impact assessment of all policies, including those that do not intend to influence health. Such HiaPs should be conducted before the implementation of a policy, but also after the implementation to evaluate the actual impact of a policy (Lhachimi 2012; Lhachimi 2013). Investigating the impact of the withdrawal of the tax may also be fruitful (Craig 2018).

## History

Protocol first published: Issue 10, 2016 Review first published: Issue 9, 2020

## Appendices

### Appendix 1. MEDLINE search strategy

12 September 2019

4235 records

1. exp Taxes/

2. exp Government Programs/ec, lj [Economics, Legislation & Jurisprudence]

3. exp Health Policy/ec, lj [Economics, Legislation & Jurisprudence]

4. exp Food Dispensers, Automatic/ec, lj, sn [Economics, Legislation & Jurisprudence, Statistics & Numerical Data]

5. exp Health Promotion/ec, lj [Economics, Legislation & Jurisprudence]

6. exp Nutrition Policy/ec, lj [Economics, Legislation & Jurisprudence]

7. exp Public Health/ec, lj [Economics, Legislation & Jurisprudence]

8. "demand elasticity".tw.

9. "policy intervention*".tw.

10. "sales tax".tw.

11. "thin subsidies".tw.

12. "vending machine*".tw.

13. budget.tw.

14. excise.tw.

15. fiscal.tw.

16. levied.tw.

17. levy.tw.

18. price.tw.

19. priced.tw.

20. prices.tw.

21. pricing.tw.

22. subsidy.tw.

23. subsidies.tw.

24. tax.tw.

25. taxation.tw.

26. taxed.tw.

27. taxes.tw.

28. taxing.tw.

29. OR/1‐28

30. exp Dietary Carbohydrates/

31. exp Dietary Sucrose/

32. exp High Fructose Corn Syrup/

33. "chewing gum".tw.

34. "dietary sucrose".tw.

35. (("energy dens*" or "highenergy" or "high energy" or "high‐energy" or "low energy" or chips) and (fat* or sugar* or sweet* or food or diet* or nutrition or overweight or drink* or beverage* or protein* or carbohydrate*)).tw.

36. "HED calori*".tw.

37. "HED‐calori*".tw.

38. "highcalori* food*".tw.

39. "high calori* food*".tw.

40. "high‐calori* food*".tw.

41. "lowcalori* food*".tw.

42. "low calori* food*".tw.

43. "low‐calori* food*".tw.

44. "ice cream*".tw.

45. "unhealthy food*".tw.

46. bakery.tw.

47. biscuit*.tw.

48. cacao.tw.

49. cake*.tw.

50. calorie*.tw.

51. candy.tw.

52. candies.tw.

53. bonbon*.tw.

54. chocolate*.tw.

55. confectionar*.tw.

56. cookie*.tw.

57. isoglucose.tw.

58. jam.tw.

59. jelly.tw.

60. jellies.tw.

61. liquorice.tw.

62. macronutrient*.tw.

63. maltose.tw.

64. marmalade.tw.

65. marzipan.tw.

66. pastr*.tw.

67. sucrose.tw.

68. sugar.tw.

69. sugars.tw.

70. sugary.tw.

71. sweet*.tw.

72. exp Butter/

73. exp Dietary Fats/

74. exp Energy Intake/

75. exp Fast Foods/

76. exp Margarine/

77. exp Plant Oils/ec [Economics]

78. "fastfood*".tw.

79. "fast food*".tw.

80. "fast‐food*".tw.

81. "fattening‐food*".tw.

82. "fattening food*".tw.

83. "fried food*".tw.

84. (coconut OR cooking OR palm OR vegetable OR soya OR soybean OR rapeseed OR linseed OR sunflower OR sesame OR peanut OR groundnut OR copra OR babassu OR olive OR thistle ADJ Oil).tw.

85. "salty‐snack*".tw.

86. "salty snack*".tw.

87. "snack food*".tw.

88. "snack‐food*".tw.

89. "takeaway food*".tw.

90. "takeaway‐food*".tw.

91. "take away food*".tw.

92. "take away‐food*".tw.

93. "take‐away food*".tw.

94. "take‐away‐food*".tw.

95. "whole milk".tw.

96. burger*.tw.

97. butter.tw.

98. cheese.tw.

99. cream.tw.

100. crisps.tw.

101. (egg AND (fat* or sugar* or sweet* or food or diet* or nutrition or overweight or drink* or beverage* or protein* or carbohydrate*)).tw.

102. (eggs AND (fat* or sugar* or sweet* or food or diet* or nutrition or overweight or drink* or beverage* or protein* or carbohydrate*)).tw.

103. (fat AND (Food* or diet* or nutrition or nutrient or eat* or meal* or oil* or carbohydrate* or protein* or obesity or obese)).tw.

104. (fatty AND (Food* or diet* or nutrition or nutrient or eat* or meal* or oil* or carbohydrate* or protein* or obesity or obese)).tw.

105. fats.tw.

106. fattening.tw.

107. fries.tw.

108. ghee.tw.

109. lard.tw.

110. margarine.tw.

111. mono‐unsat*.tw.

112. monounsat*.tw.

113. omega3.tw.

114. "omega 3".tw.

115. omega‐3.tw.

116. pizza.tw.

117. polyunsat*.tw.

118. poly‐unsat*.tw.

119. sausage*.tw.

120. suet.tw.

121. exp Carbonated Beverages/

122. exp Food Preferences/

123. exp Food Habits/

124. "caloric‐drink*".tw.

125. "caloric drink*".tw.

126. "carbonated‐beverage*".tw.

127. "carbonated beverage*".tw.

128. "carbonated‐drink*".tw.

129. "carbonated drink*".tw.

130. "energy‐drink*".tw.

131. "energy drink*".tw.

132. "fizzy‐drink*".tw.

133. "fizzy drink*".tw.

134. "high‐calori* drink*".tw.

135. "high calori* drink*".tw.

136. "soda pop".tw.

137. "soft‐drink*".tw.

138. "soft drink*".tw.

139. "sport‐drink*".tw.

140. "sport* drink*".tw.

141. "sport*‐drink*".tw.

142. cola.tw.

143. soda.tw.

144. SSB*.tw.

145. syrup*.tw.

146. OR/30‐145

147. 29 AND 146

148. (animals NOT (humans AND animals)).sh.

149. 147 NOT 148

### Appendix 2. Search strategies for electronic academic databases

#### Cochrane Central Register of Controlled Trials (CENTRAL) via Wiley (1948 to present)

9 October 2019

425 records

#1. MeSH descriptor: [Taxes] explode all trees

#2. MeSH descriptor: [Government Programs] explode all trees and with qualifier(s): [Economics ‐ EC, Legislation & jurisprudence ‐ LJ]

#3. MeSH descriptor: [Health Policy] explode all trees and with qualifier(s): [Economics ‐ EC, Legislation & jurisprudence ‐ LJ]

#4. MeSH descriptor: [Food Dispensers, Automatic] explode all trees

#5. MeSH descriptor: [Health Promotion] explode all trees and with qualifier(s): [Economics ‐ EC, Legislation & jurisprudence ‐ LJ]

#6. MeSH descriptor: [Nutrition Policy] explode all trees and with qualifier(s): [Economics ‐ EC, Legislation & jurisprudence ‐ LJ]

#7. MeSH descriptor: [Public Health] explode all trees and with qualifier(s): [Economics ‐ EC, Legislation & jurisprudence ‐ LJ]

#8. "demand elasticity"

#9. "policy intervention*"

#10. "thin subsidies"

#11. "vending machine*"

#12. budget

#13. excise

#14. fiscal

#15. levied

#16. levy

#17. price

#18. priced

#19. prices

#20. pricing

#21. subsidy

#22.subsidies

#23.tax

#24. taxation

#25. taxed

#26. taxes

#27. taxing

#28. #1 or #2 or #3 or #4 or #5 or #6 or #7 or #8 or #9 or #10 or #11 or #12 or #13 or #14 or #15 or #16 or #17 or #18 or #19 or #20 or #21 or #22 or #23 or #24 or #25 or #26 or #27

#29. MeSH descriptor: [Dietary Carbohydrates] explode all trees

#30. MeSH descriptor: [Dietary Sucrose] explode all trees

#31. "chewing gum"

#32. "dietary sucrose"

#33. "energy dens*"

#34. "highenergy"

#35. "high energy"

#36. "high‐energy"

#37. "low energy"

#38. chips

#39. "highcalori* food*"

#40. "high calori* food*"

#41. "high‐calori* food*"

#42. "low‐calori* food*"

#43. "ice cream*"

#44. "unhealthy food*"

#45. bakery

#46. biscuit*

#47. cacao

#48. cake*

#49. calorie*

#50. candy

#51. candies

#52. bonbon*

#53. chocolate*

#54. confectionar*

#55. cookie*

#56. isoglucose

#57. jam

#58. jelly

#59. jellies

#60. liquorice

#61. macronutrient*

#62. maltose

#63. marmalade

#64. marzipan

#65. pastr*

#66. sucrose

#67. sugar

#68. sugars

#69. sugary

#70. sweet*

#71. MeSH descriptor: [Butter] explode all trees

#72. MeSH descriptor: [Dietary Fats] explode all trees

#73. MeSH descriptor: [Energy Intake] explode all trees

#74. MeSH descriptor: [Fast Foods] explode all trees

#75. MeSH descriptor: [Margarine] explode all trees

#76. MeSH descriptor: [Plant Oils] explode all trees

#77. "fastfood*"

#78. "fast food*"

#79. "fast‐food*"

#80. "fattening‐food*"

#81. "fattening food*"

#82. "fried food*"

#83. "coconut oil"

#84. "cooking oil"

#85. "palm oil"

#86. "vegetable oil"

#87. "soya oil"

#88. "soybean oil"

#89. "rapeseed oil"

#90. "linseed oil"

#91. "sunflower oil"

#92. "sesame oil"

#93. "peanut oil"

#94. "groundnut oil"

#95. "copra oil"

#96. "babassu oil"

#97. "olive oil"

#98. "thistle oil"

#99. "salty‐snack*"

#100. "salty snack*"

#101. "snack food*"

#102. "snack‐food*"

#103. "takeaway food*"

#104. "takeaway‐food*"

#105. "take away food*"

#106. "take away‐food*"

#107. "take‐away food*"

#108. "take‐away‐food*"

#109. "whole milk"

#110. burger*

#111. butter

#112. cream

#113. crisps

#114. egg

#115. eggs

#116. fat

#117. fatty

#118. fats

#119. fries

#120. lard

#121. mono‐unsat*

#122. monounsat*

#123. omega3

#124. "omega 3"

#125. omega‐3

#126. polyunsat*

#127. poly‐unsat*

#128. sausage*

#129. suet

#130. MeSH descriptor: [Carbonated Beverages] explode all trees

#131. MeSH descriptor: [Food Preferences] explode all trees

#132. MeSH descriptor: [Food Habits] explode all trees

#133. "caloric‐drink*"

#134. "caloric drink*"

#135. "carbonated‐beverage*"

#136. "carbonated beverage*"

#137. "carbonated‐drink*"

#138. "carbonated drink*"

#139. "energy‐drink*"

#140. "energy drink*"

#141. "fizzy‐drink*"

#142. "fizzy drink*"

#143. "high‐calori* drink*"

#144. "high calori* drink*"

#145. "soda pop"

#146. "soft‐drink*"

#147. "soft drink*"

#148. "sport‐drink*"

#149. "sport* drink*"

#150. "sport*‐drink*"

#151. cola

#152. soda

#153. SSB*

#154. syrup*

#155. #29 or #30 or #31 or #32 or #33 or #34 or #35 or #36 or #37 or #38 or #39 or #40 or #41 or #42 or #43 or #44 or #45 or #46 or #47 or #48 or #49 or #50 or #51 or #52 or #53 or #54 or #55 or #56 or #57 or #58 or #59 or #60 or #61 or #62 or #63 or #64 or #65 or #66 or #67 or #68 or #69 or #70 or #71 or #72 or #73 or #74 or #75 or #76 or #77 or #78 or #79 or #80 or #81 or #82 or #83 or #84 or #85 or #86 or #87 or #88 or #89 or #90 or #91 or #92 or #93 or #94 or #95 or #96 or #97 or #98 or #99 or #100 or #101 or #102 or #103 or #104 or #105 or #106 or #107 or #108 or #109 or #110 or #111 or #112 or #113 or #114 or #115 or #116 or #117 or #118 or #119 or #120 or #121 or #122 or #123 or #124 or #125 or #126 or #127 or #128 or #129 or #130 or #131 or #132 or #133 or #134 or #135 or #136 or #137 or #138 or #139 or #140 or #141 or #142 or #143 or #144 or #145 or #146 or #147 or #148 or #149 or #150 or #151 or #152 or #153 or #154

#156. #155 and #28 in Trials

#### Cochrane Database of Systematic Reviews (CDSR) via Wiley (1995 to present)

9 October 2019

66 records

#1 tax or taxes or taxation

#2 food or sugar or sweet or sweets or sweetened or fast food or snacks or fat or fats or fatty or soft drinks

#3 #1 and #2

#### Excerpta Medica database (Embase) via OvidSP (1947 to present)

12 September 2019

6797 records

1. exp Tax/

2. exp government regulation/

3. "demand elasticity".tw.

4. "policy intervention*".tw.

5. "sales tax".tw.

6. "thin subsidies".tw.

7. "vending machine*".tw.

8. budget.tw.

9. excise.tw.

10. fiscal.tw.

11. levied.tw.

12. levy.tw.

13. price.tw.

14. priced.tw.

15. prices.tw.

16. pricing.tw.

17. subsidy.tw.

18. subsidies.tw.

19. tax.tw.

20. taxation.tw.

21. taxed.tw.

22. taxes.tw.

23. taxing.tw.

24. or/1‐23

25. exp carbohydrate intake/

26. exp corn syrup/

27. sugar intake/

28. sweetening agent/

29. "chewing gum".tw.

30. "dietary sucrose".tw.

31. (("energy dens*" or "highenergy" or "high energy" or "high‐energy" or "low energy" or chips) and (fat* or sugar* or sweet* or food or diet* or nutrition or overweight or drink* or beverage* or protein* or carbohydrate*)).tw.

32. "HED calori*".tw.

33. "HED‐calori*".tw.

34. "highcalori* food*".tw.

35. "high calori* food*".tw.

36. "high‐calori* food*".tw.

37. "lowcalori* food*".tw.

38. "low calori* food*".tw.

39. "low‐calori* food*".tw.

40. "ice cream*".tw.

41. "unhealthy food*".tw.

42. bakery.tw.

43. biscuit*.tw.

44. cacao.tw.

45. cake*.tw.

46. calorie*.tw.

47. candy.tw.

48. candies.tw.

49. bonbon*.tw.

50. chocolate*.tw.

51. confectionar*.tw.

52. cookie*.tw.

53. isoglucose.tw.

54. jam.tw.

55. jelly.tw.

56. jellies.tw.

57. liquorice.tw.

58. macronutrient*.tw.

59. maltose.tw.

60. marmalade.tw.

61. marzipan.tw.

62. pastr*.tw.

63. sucrose.tw.

64. sugar.tw.

65. sugars.tw.

66. sugary.tw.

67. sweet*.tw.

68. exp butter/

69. exp fat intake/

70. exp caloric intake/

71. exp fast food/

72. exp margarine/

73. exp food preference/

74. exp milk fat/

75. "fastfood*".tw.

76. "fast food*".tw.

77. "fast‐food*".tw.

78. "fattening‐food*".tw.

79. "fattening food*".tw.

80. "fried food*".tw.

81. ((coconut or cooking or palm or vegetable or soya or soybean or rapeseed or linseed or sunflower or sesame or peanut or groundnut or copra or babassu or olive or thistle) adj Oil).tw.

82. "salty‐snack*".tw.

83. "salty snack*".tw.

84. "snack food*".tw.

85. "snack‐food*".tw.

86. "takeaway food*".tw.

87. "takeaway‐food*".tw.

88. "take away food*".tw.

89. "take away‐food*".tw.

90. "take‐away food*".tw.

91. "take‐away‐food*".tw.

92. "whole milk".tw.

93. burger*.tw.

94. butter.tw.

95. cheese.tw.

96. cream.tw.

97. crisps.tw.

98. (egg and (fat* or sugar* or sweet* or food or diet* or nutrition or overweight or drink* or beverage* or protein* or carbohydrate*)).tw.

99. (eggs and (fat* or sugar* or sweet* or food or diet* or nutrition or overweight or drink* or beverage* or protein* or carbohydrate*)).tw.

100. (fat and (Food* or diet* or nutrition or nutrient or eat* or meal* or oil* or carbohydrate* or protein* or obesity or obese)).tw.

101. (fatty and (Food* or diet* or nutrition or nutrient or eat* or meal* or oil* or carbohydrate* or protein* or obesity or obese)).tw.

102. fats.tw.

103. fattening.tw.

104. fries.tw.

105. ghee.tw.

106. lard.tw.

107. margarine.tw.

108. mono‐unsat*.tw.

109. monounsat*.tw.

110. omega3.tw.

111. "omega 3".tw.

112. omega‐3.tw.

113. pizza.tw.

114. polyunsat*.tw.

115. poly‐unsat*.tw.

116. sausage*.tw.

117. suet.tw.

118. "caloric‐drink*".tw.

119. "caloric drink*".tw.

120. "carbonated‐beverage*".tw.

121. "carbonated beverage*".tw.

122. "carbonated‐drink*".tw.

123. "carbonated drink*".tw.

124. "energy‐drink*".tw.

125. "energy drink*".tw.

126. "fizzy‐drink*".tw.

127. "fizzy drink*".tw.

128. "high‐calori* drink*".tw.

129. "high calori* drink*".tw.

130. "soda pop".tw.

131. "soft‐drink*".tw.

132. "soft drink*".tw.

133. "sport‐drink*".tw.

134. "sport* drink*".tw.

135. "sport*‐drink*".tw.

136. cola.tw.

137. soda.tw.

138. SSB*.tw.

139. syrup*.tw.

140. exp dietary intake/

141. or/25‐140

142. 24 and 141

#### PsycINFO via OvidSP (1887 to present)

9 October 2019

1978 records

1. exp Taxation/

2. exp Policy Making/

3. exp Government Programs/

4. exp Government Policy Making/

5. "demand elasticity".tw.

6. "policy intervention*".tw.

7. "sales tax".tw.

8. "thin subsidies".tw.

9. "vending machine*".tw.

10. budget.tw.

11. excise.tw.

12. fiscal.tw.

13. levied.tw.

14. levy.tw.

15. price.tw.

16. priced.tw.

17. prices.tw.

18. pricing.tw.

19. subsidy.tw.

20. subsidies.tw.

21. tax.tw.

22. taxation.tw.

23. taxed.tw.

24. taxes.tw.

25. taxing.tw.

26. or/1‐25

27. exp Carbohydrates/

28. exp Food Intake/

29. exp Sugars/

30. "chewing gum".tw.

31. "dietary sucrose".tw.

32. (("energy dens*" or "highenergy" or "high energy" or "high‐energy" or "low energy" or chips) and (fat* or sugar* or sweet* or food or diet* or nutrition or overweight or drink* or beverage* or protein* or carbohydrate*)).tw.

33. "HED calori*".tw.

34. "HED‐calori*".tw.

35. "highcalori* food*".tw.

36. "high calori* food*".tw.

37. "high‐calori* food*".tw.

38. "lowcalori* food*".tw.

39. "low calori* food*".tw.

40. "low‐calori* food*".tw.

41. "ice cream*".tw.

42. "unhealthy food*".tw.

43. bakery.tw.

44. biscuit*.tw.

45. cacao.tw.

46. cake*.tw.

47. calorie*.tw.

48. candy.tw.

49. candies.tw.

50. bonbon*.tw.

51. chocolate*.tw.

52. confectionar*.tw.

53. cookie*.tw.

54. isoglucose.tw.

55. jam.tw.

56. jelly.tw.

57. jellies.tw.

58. liquorice.tw.

59. macronutrient*.tw.

60. maltose.tw.

61. marmalade.tw.

62. marzipan.tw.

63. pastr*.tw.

64. sucrose.tw.

65. sugar.tw.

66. sugars.tw.

67. sugary.tw.

68. sweet*.tw.

69. exp Eating Behavior/

70. exp Fast Food/

71. exp Fatty Acids/

72. "fastfood*".tw.

73. "fast food*".tw.

74. "fast‐food*".tw.

75. "fattening‐food*".tw.

76. "fattening food*".tw.

77. "fried food*".tw.

78. ((coconut or cooking or palm or vegetable or soya or soybean or rapeseed or linseed or sunflower or sesame or peanut or groundnut or copra or babassu or olive or thistle) adj Oil).tw.

79. "salty‐snack*".tw.

80. "salty snack*".tw.

81. "snack food*".tw.

82. "snack‐food*".tw.

83. "takeaway food*".tw.

84. "takeaway‐food*".tw.

85. "take away food*".tw.

86. "take away‐food*".tw.

87. "take‐away food*".tw.

88. "take‐away‐food*".tw.

89. "whole milk".tw.

90. burger*.tw.

91. butter.tw.

92. cheese.tw.

93. cream.tw.

94. crisps.tw.

95. (egg and (fat* or sugar* or sweet* or food or diet* or nutrition or overweight or drink* or beverage* or protein* or carbohydrate*)).tw.

96. (eggs and (fat* or sugar* or sweet* or food or diet* or nutrition or overweight or drink* or beverage* or protein* or carbohydrate*)).tw.

97. (fat and (Food* or diet* or nutrition or nutrient or eat* or meal* or oil* or carbohydrate* or protein* or obesity or obese)).tw.

98. (fatty and (Food* or diet* or nutrition or nutrient or eat* or meal* or oil* or carbohydrate* or protein* or obesity or obese)).tw.

99. fats.tw.

100. fattening.tw.

101. fries.tw.

102. ghee.tw.

103. lard.tw.

104. margarine.tw.

105. mono‐unsat*.tw.

106. monounsat*.tw.

107. omega3.tw.

108. "omega 3".tw.

109. omega‐3.tw.

110. pizza.tw.

111. polyunsat*.tw.

112. poly‐unsat*.tw.

113. sausage*.tw.

114. suet.tw.

115. exp "Beverages (Nonalcoholic)"/

116. exp Food Preferences/

117. "caloric‐drink*".tw.

118. "caloric drink*".tw.

119. "carbonated‐beverage*".tw.

120. "carbonated beverage*".tw.

121. "carbonated‐drink*".tw.

122. "carbonated drink*".tw.

123. "energy‐drink*".tw.

124. "energy drink*".tw.

125. "fizzy‐drink*".tw.

126. "fizzy drink*".tw.

127. "high‐calori* drink*".tw.

128. "high calori* drink*".tw.

129. "soda pop".tw.

130. "soft‐drink*".tw.

131. "soft drink*".tw.

132. "sport‐drink*".tw.

133. "sport* drink*".tw.

134. "sport*‐drink*".tw.

135. cola.tw.

136. soda.tw.

137. SSB*.tw.

138. syrup*.tw.

139. or/27‐138

140. 26 and 139

#### Current Contents Medicine Database of German and German‐Language Journals (CCMed) via LIVIVO (2000 to present)

10 October 2019

38 records

(((tax OR taxes OR taxation) AND (food OR sugar OR sweet OR sweets OR sweetened OR "fast food" OR snacks OR fat OR fats OR fatty OR "soft drinks" OR "soft drink")) OR ((Steuer OR Steuern OR Besteuerung) AND (Essen OR Lebensmittel OR Zucker OR Süßigkeit OR Süßigkeiten OR gesüßt OR Fastfood OR Snacks OR Fett OR Fette OR fetthaltig OR Süßgetränk OR Süßgetränke OR Softdrink OR Softdrinks))) DB=CCMED

#### Latin American and Caribbean Health Sciences (LILACS) via BIREME/VHL (1982 to present)

12 September 2019

95 records

tax or taxes or taxation or policy making [Words] and food or sugar or sweet or sweets or sweetened or fast food or snacks or fat or fats or fatty or soft drinks [Words]

#### EconLit via EBSCO (1969 to present)

9 October 2019

4142 records

S1. SU Taxes

S2. SU Government Programs

S3. SU Health Policy

S4. SU Health Promotion

S5. SU Nutrition Policy

S6. SU Public Health

S7. TX "demand elasticity"

S8. TX "policy intervention*"

S9. TX "sales tax"

S10. TX "thin subsidies"

S11. TX "vending machine*"

S12. TX budget

S13. TX excise

S14. TX fiscal

S15. TX levied

S16. TX levy

S17. TX price

S18. TX priced

S19. TX prices

S20. TX pricing

S21. TX subsidy

S22. TX subsidies

S23. TX tax

S24. TX taxation

S25. TX taxed

S26. TX taxes

S27. TX taxing

S28. S1 OR S2 OR S3 OR S4 OR S5 OR S6 OR S7 OR S8 OR S9 OR S10 OR S11 OR S12 OR S13 OR S14 OR S15 OR S16 OR S17 OR S18 OR S19 OR S20 OR S21 OR S22 OR S23 OR S24 OR S25 OR S26 OR S27

S29. TX "chewing gum"

S30. TX "dietary sucrose"

S31. TX (("energy dens*" or "highenergy" or "high energy" or "high‐energy" or "low energy" or chips) and (fat* or sugar* or sweet* or food or diet* or nutrition or overweight or drink* or beverage* or protein* or carbohydrate*))

S32. TX "high calori* food*"

S33. TX "high‐calori* food*"

S34. TX "low calori* food*"

S35. TX "low‐calori* food*"

S36. TX "ice cream*"

S37. TX "unhealthy food*"

S38. TX bakery

S39. TX biscuit*

S40. TX cacao

S41. TX cake*

S42. TX calorie*

S43. TX candy

S44. TX candies

S45. TX bonbon*

S46. TX chocolate*

S47. TX confectionar*

S48. TX cookie*

S49. TX isoglucose

S50. TX jam

S51. TX jelly

S52. TX jellies

S53. TX macronutrient*

S54. TX pastr*

S55. TX sucrose

S56. TX sugar

S57. TX sugars

S58. TX sugary

S59. TX sweet*

S60. SU Butter

S61. SU Fast Foods

S62. TX "fastfood*"

S63. TX "fast food*"

S64. TX "fast‐food*"

S65. TX "fattening‐food*"

S66. TX "fattening food*"

S67. TX "fried food*"

S68. TX "coconut oil"

S69. TX "cooking oil"

S70. TX "palm oil"

S71. TX "vegetable oil"

S72. TX "soya oil"

S73. TX "soybean oil"

S74. TX "rapeseed oil"

S75. TX "linseed oil"

S76. TX "sunflower oil"

S77. TX "peanut oil"

S78. TX "groundnut oil"

S79. TX "olive oil"

S80. TX "salty‐snack*"

S81. TX "salty snack*"

S82. TX "snack food*"

S83. TX "snack‐food*"

S84. TX "take away food*"

S85. TX "take away‐food*"

S86. TX "take‐away food*"

S87. TX "take‐away‐food*"

S88. TX "whole milk"

S89. TX burger*

S90. TX butter

S91. TX cheese

S92. TX cream

S93. TX crisps

S94. TX egg

S95. TX eggs

S96. TX fat

S97. TX fatty

S98. TX fats

S99. TX fattening

S100. TX fries

S101. TX ghee

S102. TX lard

S103. TX margarine

S104. TX monounsat*

S105. TX omega3

S106. TX "omega 3"

S107. TX omega‐3

S108. TX pizza

S109. TX polyunsat*

S110. TX sausage*

S111. TX suet

S112. SU Carbonated Beverages

S113. SU Food Preferences

S114. TX Food Habits

S115. TX "carbonated‐beverage

S116. TX "carbonated beverage*"

S117. TX "energy‐drink*"

S118. TX "energy drink*"

S119. TX "high‐calori* drink*"

S120. TX "high calori* drink*"

S121. TX "soda pop"

S122. TX "soft‐drink*"

S123. TX "soft drink*"

S124. TX "sport* drink*"

S125. TX "sport*‐drink*"

S126. TX cola

S127. TX soda

S128. TX SSB*

S129. TX syrup*

S130. S29 OR S30 OR S31 OR S32 OR S33 OR S34 OR S35 OR S36 OR S37 OR S38 OR S39 OR S40 OR S41 OR S42 OR S43 OR S44 OR S45 OR S46 OR S47 OR S48 OR S49 OR S50 OR S51 OR S52 OR S53 OR S54 OR S55 OR S56 OR S57 OR S58 OR S59 OR S60 OR S61 OR S62 OR S63 OR S64 OR S65 OR S66 OR S67 OR S68 OR S69 OR S70 OR S71 OR S72 OR S73 OR S74 OR S75 OR S76 OR S77 OR S78 OR S79 OR S80 OR S81 OR S82 OR S83 OR S84 OR S85 OR S86 OR S87 OR S88 OR S89 OR S90 OR S91 OR S92 OR S93 OR S94 OR S95 OR S96 OR S97 OR S98 OR S99 OR S100 OR S101 OR S102 OR S103 OR S104 OR S105 OR S106 OR S107 OR S108 OR S109 OR S110 OR S111 OR S112 OR S113 OR S114 OR S115 OR S116 OR S117 OR S118 OR S119 OR S120 OR S121 OR S122 OR S123 OR S124 OR S125 OR S126 OR S127 OR S128 OR S129

S131. S28 AND S130

#### Campbell Library via Campbell Collaboration (2004 to present)

9 October 2019

23 records

'tax or taxes or taxation' IN All text and 'food* or sugar* or sweet or sweets or sweetened or fast food or snacks or fat or fats or fatty or soft drinks' in All text

#### Food Science and Technology Abstracts (FSTA) via OvidSP (1969 to present)

14 October 2019

1325 records

1 exp pricing/ or taxation/

2 ("tax" or "taxation" or "taxed" or "taxes" or "taxing" or "levy" or "levied").mp.

3 (("price" or "pricing" or "prices") adj2 "change?").mp.

4 (("price" or "pricing" or "prices") adj2 ("intervention?" or "experiment?")).mp.

5 ("sugar?" or "added food?" or "fat?" or "saturated" or "caloric" or "soft drink?" or "SSB?" or "sweetened beverage?" or "soft drink?" or "carbonated drink?").mp.

6 or/1‐4

7 5 and 6

#### Cumulative Index to Nursing and Allied Health Literature (CINAHL) via EBSCO (1937 to present)

12 September 2019

2792 records

S2 (MH "Government Programs+")

S3 (MH "Health Promotion+")

S4 (MH "Nutrition Policy+")

S5 TX "demand elasticity" OR TX "policy intervention*" OR TX "sales tax" OR TX "thin subsidies" OR TX "vending machine*" OR TX budget OR TX excise OR TX fiscal OR TX levied OR TX levy OR TX price OR TX priced

S6 TX prices OR TX pricing OR TX subsidy OR TX subsidies OR TX tax OR TX taxation OR TX taxed OR TX taxes OR TX taxing

S7 S1 OR S2 OR S3 OR S4 OR S5 OR S6

S8 (MH "Dietary Carbohydrates+")

S9 (MH "Dietary Sucrose")

S10 (MM "High Fructose Corn Syrup")

S11 TX "chewing gum" OR TX ( (("energy dens*" or "highenergy" or "high energy" or "high‐energy" or "low energy" or chips) and (fat* or sugar* or sweet* or food or diet* or nutrition or overweight or drink* or beverage* or protein* or carbohydrate*)) ) OR TX "high calori* food*" OR TX "high‐calori* food*" OR TX "low calori* food*" OR TX "low‐calori* food*" OR TX "ice cream*" OR TX "unhealthy food*" OR TX bakery OR TX biscuit* OR TX cacao OR TX cake*

S12 TX calorie* OR TX candy OR TX bonbon* OR TX chocolate* OR TX confectionar* OR TX cookie* OR TX isoglucose OR TX jam OR TX jelly OR TX jellies OR TX macronutrient* OR TX pastr*

S13 TX sucrose OR TX sugar OR TX sugars OR TX sugary OR TX sweet* OR TX Butter OR TX "fastfood*" OR TX "fast food*" OR TX "fast‐food*" OR TX "fattening‐food*" OR TX "fattening food*" OR TX "fried food*"

S14 TX "coconut oil" OR TX "cooking oil" OR TX "palm oil" OR TX "vegetable oil" OR TX "soya oil" OR TX "soybean oil" OR TX "rapeseed oil" OR TX "linseed oil" OR TX "sunflower oil" OR TX "peanut oil" OR TX "groundnut oil" OR TX "olive oil"

S15 TX "salty‐snack*" OR TX "salty snack*" OR TX "snack food*" OR TX "snack‐food*" OR TX "take away food" OR TX "take away‐food*" OR TX "take‐away food" OR TX "take‐away‐food*" OR TX "whole milk" OR TX burger* OR TX cheese OR TX cream

S16 TX crisps OR TX ( (egg and (fat* or sugar* or sweet* or food or diet* or nutrition or overweight or drink* or beverage* or protein* or carbohydrate*)) ) OR TX ( (eggs and (fat* or sugar* or sweet* or food or diet* or nutrition or overweight or drink* or beverage* or protein* or carbohydrate*)) ) OR TX ( (fat and (Food* or diet* or nutrition or nutrient or eat* or meal* or oil* or carbohydrate* or protein* or obesity or obese)) ) OR TX ( (fatty and (Food* or diet* or nutrition or nutrient or eat* or meal* or oil* or carbohydrate* or protein* or obesity or obese)) ) OR TX fats OR TX fattening OR TX fries OR TX ghee OR TX lard OR TX margarine OR TX mono‐unsat*

S17 TX monounsat* OR TX omega3 OR TX "omega 3" OR TX omega‐3 OR TX pizza OR TX polyunsat* OR TX sausage* OR TX suet

S18 (MM "Carbonated Beverages")

S19 (MM "Food Preferences")

S20 (MM "Food Habits")

S21 TX "caloric‐drink*" OR TX "caloric drink*" OR TX "carbonated‐beverage*" OR TX carbonated beverages OR TX "carbonated‐drink*".

S22 TX "carbonated drink*" OR TX "energy‐drink*" OR TX "energy drink*" OR TX "fizzy‐drink*" OR TX "fizzy drink*" OR TX "high‐calori* drink*" OR TX "high calori* drink*" OR TX "soda pop" OR TX "soft‐drink*" OR TX "soft drink*" OR TX "sport‐drink*" OR TX "sport* drink*"

S23 TX "sport*‐drink*" OR TX cola OR TX soda OR TX SSB* OR TX syrup*

S24 S8 OR S9 OR S10 OR S11 OR S12 OR S13 OR S14 OR S15 OR S16 OR S17 OR S18 OR S19 OR S20 OR S21 OR S22 OR S23

S25 S7 AND S24

S26 Restrict S25 to Academic Journals and Dissertations

#### Web of Science (SCI‐EXPANDED, SSCI, A&HCI, CPCI‐S, CPCI‐SSH, ESCI, CCR‐EXPANDED, IC) via Clarivate Analytics (1900 to present)

12 September 2019

1364 records

TOPIC: (((("TAX" OR "TAXATION" OR "TAXED" OR "TAXES" OR "TAXING" OR "LEVY" OR "LEVIED") OR (("PRICE" OR "PRICING" OR "PRICES") NEAR "CHANGE$") OR (("PRICE" OR "PRICING" OR "PRICES") NEAR ("INTERVENTION$" OR "EXPERIMENT$"))))) AND TOPIC: ((("SUGAR$" OR "ADDED FOOD$" OR "FAT$" OR "SATURATED" OR "CALORIC" OR "SOFT DRINK$" OR "SSB$" OR "SWEETENED BEVERAGE$" OR "SOFT DRINK$" OR "CARBONATED DRINK$")))

Timespan: All years. Indexes: SCI‐EXPANDED, SSCI, A&HCI, CPCI‐S, CPCI‐SSH, BKCI‐S, BKCI‐SSH, ESCI, CCR‐EXPANDED, IC

### Appendix 3. Search strategies for grey literature databases

#### ProQuest Dissertations & Theses Database (PQDT): UK and Ireland via ProQuest

9 September 2019

105 records

(ab(tax) OR ti(tax) OR ab(taxes) OR ti(taxes) OR ab(taxation) OR ti(taxation) ab(budget*) OR ti(budget*) OR ab(excise) or ti(excise)) AND (ab(sugar*) OR ti(sugar*) OR ab(sweet*) OR ti(sweet*) OR ab("fast food*") OR ti("fast food*") OR ab(snack*) OR ti(snack*) OR ab(fat) OR ti(fat) OR ab(fatty) OR ti(fatty) OR ab(fats) OR ti(fats) OR ab("soft drink*") OR ti("soft drink*") OR ab(beverage*) OR ti(beverage*) OR ab(food*) OR ti(food*))

#### System for Information on Grey Literature in Europe – OpenGrey via OpenGrey

9 September 2019

33 records

((tax OR taxes OR taxation) AND (food OR sugar OR sweet OR sweets OR sweetened OR fast food OR snacks OR fat OR fats OR fatty OR "soft drinks" OR "soft drink"))

#### The Directory of Open Access Repositories – OpenDOAR via OpenDOAR

12 December 2016 (database not accessible in subsequent searches)

71 records

(tax OR taxes OR taxation) AND (food OR sugar OR sweet OR sweets OR sweetened OR fast food OR snacks OR fat OR fats OR fatty OR "soft drinks" OR "soft drink")

#### EconPapers via REPEC

14 October 2019

90 records

(tax OR taxes OR taxation) AND (food OR sugar OR sweet OR sweets OR sweetened OR fast food OR snacks OR fat OR fats OR fatty OR "soft drinks" OR "soft drink")

#### Social Science Research Network – SSRN eLibrary via SSRN

14 October 2019

168 records

"sugar tax" OR sweetened OR "Nutrient‐Specific Taxes" OR "soda taxes" OR "food tax" OR "fat tax"

#### National Bureau of Economic Research (NBER) via NBER

13 October 2019

335 records

(tax OR taxes OR taxation) AND (food OR sugar OR sweet OR sweets OR sweetened OR fast food OR snacks OR fat OR fats OR fatty OR "soft drinks" OR "soft drink")

#### WHO International Clinical Trials Registry Platform (WHO ICTRP) (includes references of the ClinicalTrials.gov database)

14 October 2019

164 records

(TITLE: tax or taxation or taxed or taxes or taxing or levy or levied or price or pricing or prices) OR (INTERVENTION: tax or taxation or taxed or taxes or taxing or levy or levied or price or pricing or prices)

#### Trials Register of Promoting Health Interventions (TRoPHI) via EPPI‐Centre

11 August 2016 (free text search not accessible in subsequent searches)

41 records

Freetext (All but Authors): "tax" or "taxation" or "taxed" or "taxes" or "taxing" or "levy" or "levied" or "price" or "pricing" or "prices"

### Appendix 4. Search strategies for internet search engines

#### Google Scholar via Google

11 August 2016: 30 records

14 October 2019: 30 records

Total: 60 records

(tax OR taxes OR taxation) AND (food OR sugar OR sweet OR sweets OR sweetened OR fast food OR snacks OR fat OR fats OR fatty OR "soft drinks" OR "soft drink")

## Characteristics of studies

### Characteristics of included studies [ordered by study ID]

**Table:** Bodker 2015 (supporting study)

***Study characteristics***		
Methods	**Study design:** uncontrolled before‐after (UBA) **Study location/setting:** Denmark **Timing:** retrospective **Allocation to group:** not applicable (study without control group) **Database:** Nielsen Scan Track **Year of study:** 2015 **Duration of the study:** January 2010 to July 2013 (112 weeks; only the first 28 weeks of each calendar year were included in the study). **Pre‐intervention:** January 2010 to July 2010; January 2011 to July 2011 (56 weeks) **Intervention:** January 2012 to July 2012 (28 weeks) **Post‐intervention:** January 2013 to July 2013 (28 weeks) **Unit of analysis:** supermarkets (data aggregated to the whole nation)	
Participants	**Study population:** shoppers from all Danish outlet chains (except two discount chains); the study did not include individual participant data. **Country:** Denmark **Age:** all ages **Sex:** all sexes **Socioeconomic characteristics:** different socioeconomic groups **Eligibility criteria:** not applicable **Inclusion criteria:** not applicable **Recruitment:** not applicable **Equity considerations:** not applicable	
Interventions	**Intervention:** Danish tax on saturated fat, imposed on food items with more than 2.3% saturated fat (e.g. not covering regular drinking milk or yoghurts). The tax level was set DKK 16 (approximately USD 2.90) for each kilogram of saturated fat.	
Outcomes	**Primary outcome** None (see notes) **Secondary outcome** Demand: total sales for different type of food products (in metric tonnes) for the first 24 weeks of the years 2010 to 2014.	
Notes	This was used as a supporting study in our review. The study contains tables showing estimates for average daily consumption of different types of fat in kilojoules/person and average proportions of energy intake out of total energy intake for different types of fats; however, those estimates are based on combining heterogeneous data sources in addition to the study data and, hence, are not directly observed ( a modelling study). We contacted the corresponding author to request details concerning clarifications and additional data (e.g. sample size, measures of statistical precision), however, the author was not in a position to provide these data. **Funding sources:** Danish Health Foundation (2013B228) **Conflicts of interest:** the authors reported to have no conflicts of interest.	
***Risk of bias***		
**Bias**	**Authors' judgement**	**Support for judgement**
EPHPP Criteria	Unclear risk	**Component Ratings** **A) Selection bias** Q1) Are the individuals selected to participate in the study likely to be representative of the target population? *Somewhat likely (automatically collected sales**data covering all supermarkets except 2 discount chains)* Q2) What percentage of selected individuals agreed to participate? *Not applicable**(automatically collected sales data independent of the intervention)* **Rating (section A): moderate** **B) Study design** Indicate the study design: *uncontrolled before‐after* Was the study described as randomized? *No* **Rating (section B): weak** **C) Confounders** Q1) Were there important differences between groups prior to the intervention? *Not applicable* Q2) If YES, indicate the percentage of relevant confounders that were controlled (either in design (e.g. stratification, matching) or analysis)? *Not applicable* **Rating (section C): weak** **D) Blinding** Q1) Was (were) the outcome assessor(s) aware of the intervention or exposure status of participants? *No (automatically collected sales data independent of the intervention)* Q2) Were the study participants aware of the research question? *No (automatically collected sales data independent of the intervention)* **Rating (section D): strong** **E) Data collection methods** Q1) Were data collection tools shown to be valid? *Unknown* Q2) Were data collection tools shown to be reliable? *Unknown* **Rating (section E): weak** **F) Withdrawals and dropouts** Q1) Were withdrawals and dropouts reported in terms of numbers and/or reasons per group? *Not applicable* Q2) Indicate the percentage of participants completing the study. (If the percentage differs by groups, record the lowest). *Not applicable* **Rating (section F): weak** **G) Intervention integrity** Q1) What percentage of participants received the allocated intervention or exposure of interest? *80% to 100%* Q2) Was the consistency of the intervention measured? *Not applicable* Q3) Is it likely that subjects received an unintended intervention (contamination or cointervention) that may influence the results? *No* **H) Analyses** Q1) Indicate the unit of allocation. *Community (nation)* Q2) Indicate the unit of analysis. *Community, because aggregated to the whole nation* Q3) Are the statistical methods appropriate for the study design? No (primary outcomes); *Yes (secondary outcome).* Q4) Is the analysis performed by intervention allocation status (i.e. intention‐to‐treat) rather than the actual intervention received? *Not applicable* **Global rating for this paper: weak**

**Table:** Jensen 2013

***Study characteristics***		
Methods	**Study design:** interrupted time series (ITS) complying with the Effective Practice and Organisation of Care (EPOC) group (EPOC 2009; EPOC 2013) criteria, i.e. at least three time points before and after the intervention; a clearly identified intervention point; accounting of a possible time trend and possible seasonal effects; and accounting for possible autocorrelation. The precise model specification is a Tobit model (to account for the possibility of zero consumption) with correlated random‐effects and defining season dummies for each month and for Christmas, and also a linear and quadratic overall time trend. **Study location/setting:** Denmark **Timing:** retrospective **Allocation to group:** not applicable (study without control group) **Database:** GfK Panel Service Denmark **Year of study:** 2013 **Duration of the study:** 1 January 2008 to 1 July 2012 (235 weeks) **Pre‐intervention:** 1 January 2008 to 30 September 2011 (196 weeks) **Intervention:** 1 October 2011 until the end of the study (39 weeks) ‐ only selected food products, i.e. butter blends, margarine, and oil.	
Participants	**Study population:** approximately 2000 households (the study did not include individual participant data) **Country:** Denmark **Age:** all ages **Sex:** all sexes **Socioeconomic characteristics:** different socioeconomic groups **Eligibility criteria:** not applicable **Inclusion criteria:** the study was based on a commercial Danish household panel for the purpose of market research aiming to be representative for the whole population. However, the study does contain more detailed information on recruitment. **Equity considerations:** only households willing to participate in recording their purchases are included; this may exclude vulnerable parts of the population such as the homeless.	
Interventions	**Intervention:** Danish tax on saturated fat covering food items with more than 2.3% saturated fat (e.g. not covering regular drinking milk or yoghurts). The tax level was set at DKK 16 (approximately USD 2.90) for each kilogram of saturated fat.	
Outcomes	**Primary Outcome** Consumption: changes in total fat intake per household in grams per week (calculations based on the four mentioned food products) **Secondary Outcome** None reported (see notes)	
Notes	We contacted the corresponding author to request details concerning clarifications and additional data (e.g. sample size, measures of statistical precision), however the author was not in position to provide these data. **Funding sources:** not reported. **Conflicts of interest:** authors did not provide any information about conflicts of interests. The study contains tables about changes in the demand of the three food products; however, these estimates are based on a different, restrictive statistical model that does not account for the full tax effect (Jensen 2020; Jensen 2020a).	
***Risk of bias***		
**Bias**	**Authors' judgement**	**Support for judgement**
Blinding of participants and personnel (performance bias) All outcomes	Low risk	Although the intervention was not blinded, participants were not aware that their data were used to investigate the effect of the tax.
Blinding of outcome assessment (detection bias) All outcomes	Low risk	Although the intervention was not blinded, participants were not aware that their data were used to investigate the effect of the tax.
Incomplete outcome data (attrition bias) All outcomes	Unclear risk	Authors reported that their unbalanced panel had an annual attrition/replacement level of 20%. No further analysis was provided to indicate what extent this attrition and replacement may affect outcome measures.
Selective reporting (reporting bias)	Unclear risk	The study had no published study protocol. The outcomes described in the methods section were reported, however, data were derived from databases that are permanent, commercial data collections for the purpose of market research potentially covering a wide range of products. Additionally, the study included certain food types without a clear rationale for selecting particular food products.
Intervention independent of other changes (ITS)	Low risk	The intervention was a legislative act that did not have health outcomes as a motivation. Cointerventions were reported.
Shape of effect pre‐specified (ITS)	Low risk	Shape of effect of intervention was prespecified.
Intervention unlikely to affect data collection (ITS)	Low risk	Sources and methods of data collection were the same before and after the intervention: self‐reported shopping data from a permanent consumer panel for market research.
Other bias	Unclear risk	People who self‐collect shopping data might be more price‐sensitive than the general population.

**Table:** Jensen 2015

***Study characteristics***		
Methods	**Study design:** interrupted time series (ITS) complying with the EPOC (EPOC 2009; EPOC 2013) criteria: i.e. at least three time points before and after the intervention; a clearly identified intervention point; accounting of a possible time trend and possible seasonal effects; and accounting for possible autocorrelation. The precise model specification is a linear almost ideal demand system (LAIDS), using three stage least square with fixed effects with regards to stores and defining season dummies for each month (Wooldridge 2007). **Study location/setting:** Denmark **Timing:** retrospective **Allocation to group:** not applicable (study without control group) **Number of individuals:** sales data from a balanced panel of 1293 supermarkets **Database:** Coop Danmark (Danish food retailer with 40% market share) **Year of study:** 2015 **Duration of the study:** 1 January 2010 to 31 October 2012 (148 weeks) **Pre‐intervention:** 1 January 2010 to 30 September 2011 (91 weeks) **Intervention:** 1 October 2011 (57 weeks until end of study duration) **Unit of analysis:** supermarket level	
Participants	**Study population:** Coop Denmark supermarkets **Country:** Denmark **Age:** all ages **Sex:** all sexes **Socioeconomic characteristics:** different socioeconomic groups **Eligibility criteria:** not applicable **Inclusion criteria:** all Danish supermarkets that belong to a sales co‐operative **Recruitment:** no information given **Equity considerations:** not applicable	
Interventions	**Intervention:** Danish tax on saturated fat covering food items with more than 2.3% saturated fat (e.g. not covering regular drinking milk or yoghurts). The tax level was set at DKK 16 (approximately USD 2.90) for each kilogram of saturated fat.	
Outcomes	**Primary Outcome** Consumption: monthly changes in per cent of total purchased saturated fat based on the average fat content of different food products (for minced beef, regular cream, sour cream) **Secondary Outcome** Substitution: monthly changes in percent of total purchased food products, which are rich in fats (i.e. minced beef, regular cream, sour cream)	
Notes	We contacted the corresponding author to request details concerning clarifications and additional data (e.g. sample size, measures of statistical precision), however, the author was not in position to provide this data. **Funding sources:** the authors reported that the project was part of a large research centre ‘UNIK – Food, Pharma, Fitness’ at the University of Copenhagen. The centre has obtained financial support from the Danish Ministry of Science. **Conflict of Interest:** the authors declare that they have no competing interests.	
***Risk of bias***		
**Bias**	**Authors' judgement**	**Support for judgement**
Blinding of participants and personnel (performance bias) All outcomes	Low risk	Although the intervention was not blinded, participants were not aware that their data were used to investigate the effect of the tax.
Blinding of outcome assessment (detection bias) All outcomes	Low risk	Although the intervention was not blinded, participants were not aware that their data were used to investigate the effect of the tax.
Incomplete outcome data (attrition bias) All outcomes	Unclear risk	The authors did report on the completeness of their outcome data.
Selective reporting (reporting bias)	Unclear risk	The study had no published study protocol. The outcomes described in the methods section were reported, however, data were derived from databases that are permanent, commercial data collections for the purpose of market research potentially covering a wide range of products. Additionally, the study included certain food types, without providing a clear rationale for selecting particular food products.
Intervention independent of other changes (ITS)	Low risk	The intervention was a legislative act that did not have health outcomes as a motivation. No cointerventions were reported.
Shape of effect pre‐specified (ITS)	Low risk	Shape of the effect of the intervention was prespecified.
Intervention unlikely to affect data collection (ITS)	Low risk	Sources and methods of data collection were the same before and after the intervention: sales data collected through supermarket electronic cashier.
Other bias	Low risk	We did not identify other sources of bias.

EPHPP: Effective Public Health Practice Project

### Characteristics of excluded studies [ordered by study ID]

**Table:**

Study	Reason for exclusion
Elbel 2013	Intervention was not a tax on fat content; no measurement of baseline data before the intervention
Hannan 2002	Intervention was not a tax but a subsidy of low‐fat foods.
Khan 2015	Intervention was not a tax, but evidence based on (natural) variation of milk prices and not an active price manipulation of a product.
Smed 2016	The estimates for the primary outcome measure combined heterogeneous data sources and therefore this constituted a modelling study; sales data were not reported.
Taillie 2017	Intervention was not a tax on fat content; the intervention was applied indiscriminately to high‐caloric foods in general.

## Differences between protocol and review

As reported in our protocol, we planned to plot the effect direction, to analyze reporting bias (using funnel plots), perform meta‐analysis, and do subgroup and sensitivity analysis wherever possible. However, since insufficient studies were eligible for inclusion, these methods were not implemented. We did not undertake harvest plots for narrative synthesis. We further specified our eligibility criteria on types of interventions. We excluded virtual and hypothetical interventions imitating a taxation on fat content of food if participants' purchase decisions were not binding so that they did not all result in a real purchase, or if the money was virtual or not belonging to the study participant.

We added text to the Methods section to clarify our definition of the overall risk of bias of included studies, and our criteria for appropriate analysis of interrupted time series (ITS) studies.

We did not search the website from The Obesity Society (www.obesity.org), as it was not possible to enter the search terms. The database from the website The Directory of Open Access Repositories – OpenDOAR, via OpenDOAR, was not accessible after the search on 12 December 2016. The free‐text search on the website Trials Register of Promoting Health Interventions (TRoPHI), via EPPI‐Centre, was not accessible after the search on 11 August 2016.
